# Risk Factors for Hepatocellular Carcinoma in Latino Populations in Texas: A Scoping Review

**DOI:** 10.3390/ijms27104648

**Published:** 2026-05-21

**Authors:** Lais Yuki Tuzino Kamia, Emily Gonzalez, Cassandra M. Swanson, Stephanie L. Gomez, Ariann M. Canales, Ramona Salcedo Price

**Affiliations:** Nutrition and Foods Program, School of Family and Consumer Sciences, College of Applied Arts, Texas State University, San Marcos, TX 78666, USA; guw13@txstate.edu (L.Y.T.K.); jln127@txstate.edu (E.G.); qdt10@txstate.edu (C.M.S.); slg229@txstate.edu (S.L.G.); ariannlehmann@gmail.com (A.M.C.)

**Keywords:** hepatocellular carcinoma, Hispanic/Latino populations, Texas, metabolic risk factors, environmental risk factors, genetics

## Abstract

Hepatocellular carcinoma (HCC) incidence in Texas is 45% higher than the national average, with disproportionate burden among the Hispanic/Latino population. Despite significant health disparities, comprehensive evidence on HCC risk factors specific to this population remains limited. This scoping review of 20 primarily observational studies utilized PubMed, EbscoHost, and the PRISMA-ScR checklist to map risk factors in south Texas. Results show that metabolic dysfunction, specifically diabetes and obesity, increases advanced liver disease odds by 7- to 12-fold compared to non-Hispanic groups. Environmental exposures are also significant: aflatoxin was detected in 5.7 to 7.3% of Hispanic/Latino HCC tumors, and cases demonstrated 6-fold higher odds of aflatoxin biomarkers, while alcohol contributed to 3.0% of cancers. Furthermore, PNPLA3 genetic variants exerted synergistic effects with obesity and heavy alcohol consumption. Among four intervention studies, strategies included low-dose calcium montmorillonite clay for aflatoxin reduction, community-health-worker-integrated chronic care, and hospital-based hepatitis screening. However, critical research gaps remain regarding multirisk factor interactions, toxin dose–response characterization, dietary interventions, and longitudinal data. These findings underscore the urgent need for culturally tailored, community-engaged prevention programs and ethnicity-specific HCC guidelines for the Texas Hispanic/Latino population to effectively address these rising health disparities.

## 1. Introduction

HCC, the most common type of primary liver cancer in adults, accounts for an estimated 90% of liver cancer cases worldwide [1]. Globally, liver cancer represents a major public health burden, with more than 800,000 new cases and 700,000 fatalities per year, ranking as the sixth most prevalent cancer diagnosis and the third leading cause of cancer-related mortality [2].

In the United States (U.S.), liver and intrahepatic bile duct (IHBD) cancer remains a substantial public health burden, with an age-adjusted incidence rate of 8.3 per 100,000 people in 2022, marked sex disparities (12.0 males versus 5.0 in females), and persistently poor survival [3]. Amongst the U.S. states, Texas bears a disproportionately high burden of liver and IHBD cancer across multiple epidemiologic indicators [USCS]. According to the U.S. Cancer Statistics, in 2022, the age-adjusted incidence rate of liver and IHBD cancer in Texas was 12.3 per 100,000 people, with 4095 new cases reported statewide [3]. This rate exceeds national averages, highlighting Texas as a high-burden state for liver-related malignancies [3,4]. Mortality was 140.1 deaths per 100,000 in 2023 and 5-year survival rate was 20.1% [3]. These statewide indicators demonstrate Texas as a high-burden setting for persistent challenges in cancer outcomes and provide important context for examining geographic variation in liver and IHBD cancer epidemiologic indicators across Texas counties [3]. Importantly, Texas Cancer Registry surveillance data indicate that liver and IHBD cancer is one of the few cancers in the state for which both incidence and mortality have continued to rise over recent decades, with incidence rates more than doubling and mortality increasing by approximately 43% between 1995 and 2022. These data reiterate national sex disparities with incidence and mortality rates 2–3 times higher in men than in women (incidence: 18.9 vs. 7.0 per 100,000; mortality: 12.1 vs. 5.2 per 100,000).

When isolating county-level incidence data for liver and IHBD cancer in Texas from 2017–2021, incidence is not evenly distributed [5]. Data from the National Cancer Institute and Centers for Disease Control and Prevention demonstrate that the highest rates are concentrated in south Texas, with prominent clustering along the U.S.–Mexico border region along the lower Gulf Coast [5]. Approximately 10% of Texas counties (25 of 254) fall within the highest incidence category defined as age-adjusted rates exceeding 16.3 cases per 100,000 population [6]. Further stratification by race and ethnicity reveals, that among Texas counties with reportable incidence estimates, 51 of 55 counties have liver and IHBD cancer incidence rates exceeding the U.S. Hispanic national average of 13.7 per 100,000, with 13 counties demonstrating rates at least double this benchmark [6,7]. These counties are primarily located in central and south Texas, including multiple counties along the U.S.–Mexico border [5,6]. Together, these geographic and race- and ethnicity-tailored disparities motivated the need for targeted investigation related to HCC risk within Texas Hispanic/Latino/a populations of south Texas, examined in the present systematic literature review [3,5,6]. Additionally, a recent cross-sectional trend analysis using U.S. Cancer Statistics data from 2001–2021 found that, while national HCC incidence decreased from 2018–2021, incidence trends leveled in the West South Central census division, which includes Texas, with authors reinforcing race- and ethnicity-tailored and place-specific interventions are needed to reduce persistent disparities [8].

A concerning rise is observed in Western countries, driven by metabolic-dysfunction-associated steatotic liver disease (MASLD) and alcohol-associated liver disease (ALD), with Latin America experiencing a 60% rise in obesity-related HCC since 2000 [9,10]. In the U.S., HCC incidence has increased by 3% annually since 2000, with MASLD now accounting for 21% of cases [2]. Demographically, HCC disproportionately affects men, facing a 2.5-fold higher incidence than women, driven by higher rates of viral hepatitis exposure, alcohol use, and testosterone-driven oncogenesis [9]. Ethnic minorities are also disproportionately affected; Yao et al. report the highest incidence among Hispanic individuals (12.1 per 100,000) and Asian/Pacific Islanders (11.2 per 100,000), compared to 7.2 per 100,000 in non-Hispanic White populations. Regional variations also highlight socioeconomic and healthcare access inequities: southern U.S. states exhibit 25% higher mortality rates than the national average, likely due to delayed diagnoses and limited subspecialty care [9,11].

### 1.1. Etiology and Risk Factors

HCC arises from a complex interplay of genetic, environmental, and metabolic factors. In approximately 80% of cases, HCC develops from chronic liver injury that progresses from fibrosis to cirrhosis [12]. Persistent inflammation triggers oxidative stress, DNA damage, and dysregulated repair mechanisms, fostering malignant transformation [10]. Modifiable risk factors such as alcohol misuse, poor quality diet, obesity, and diabetes contribute to at least 40% of HCC cases, highlighting the importance of prevention-focused strategies [13]. Non-viral etiologies account for a growing share of HCC cases in Western populations. MASLD, driven largely by obesity and insulin resistance, represents 6.8% of global HCC and 21% of U.S. cases [14,15]. Within this etiological framework, growing evidence suggests that observed geographic, race, and ethnicity-specific disparities in HCC are complex and multifactorial, reflecting differential exposure to established risk factors including but not limited to alcohol use, aflatoxin exposure, chronic hepatitis B and C viruses (HBV, HCV), dietary patterns, genetic susceptibility, metabolic syndrome (MetS), type 2 diabetes (T2D), and obesity [13,15,16,17].

Chronic hepatitis B and C remain the leading causes of HCC worldwide, accounting for ~70% of cases, with HBV inducing oncogenic mutations and HCV promoting chronic inflammation and fibrosis; although direct-acting antivirals have reduced HCV-related HCC, residual risk persists in cirrhotic patients [11,18]. However, in Western populations, metabolic risk factors increasingly dominate HCC etiology. Obesity increases HCC risk by driving hepatic steatosis, insulin resistance (IR), and chronic inflammation that promote MASLD progression to cirrhosis and activate oncogenic signaling pathways in hepatocytes [19,20,21]. T2D independently increases HCC risk through hyperinsulinemia, hepatic fat accumulation, and systemic inflammation, with evidence showing significantly elevated HCC risk amongst patients with MASLD/NAFLD [21,22]. MetS, characterized by central obesity, dyslipidemia, hypertension (HTN), and IR, is strongly associated with MASLD and increased HCC risk, with a meta-analysis reporting a pooled odds ratio (OR) of 1.81, particularly in high-prevalence populations such as Latino/as [13,23].

Environmental and dietary exposures further modify HCC risk. Dietary patterns influence HCC risk, with diets high in red and processed meats, saturated fats, and added sugars increasing risk, while plant-based, fiber- and antioxidant-rich diets may confer protective effects by reducing hepatic inflammation and oxidative stress [17,24]. Alcohol consumption accounts for 32–45% of HCC cases and promotes carcinogenesis through oxidative stress and fibrosis, with risk substantially amplified by heavy long-term use and synergistic interaction with viral hepatitis and genetic susceptibility such as PNPLA3 [25,26]. Additionally, chronic exposure to aflatoxin B1, a foodborne mycotoxin, increases HCC risk by inducing oncogenic mutations, particularly in the *TP53* gene, and acts synergistically with HBV infection in high-prevalence regions [15,27]. Lastly, genetic susceptibility also contributes to HCC risk, with variants such as PNPLA3 (rs738409) linked to hepatic fat accumulation, fibrosis, and increased HCC risk among Hispanic/Latino populations, and *TP53* mutations associated with aflatoxin exposure and tumor progression; despite growing interest in incorporating genetic markers into HCC risk models, genetic risk specific to Latino populations remains limited and underexplored [28,29,30].

### 1.2. Rationale for Scoping Review

Due to the high burden of liver cancer among Hispanic/Latino populations in Texas, particularly in central and south Texas, there is a critical need to examine the range of risk factors contributing to HCC in this population [6]. National declines in HCC incidence may mask important regional patterns, and recent trend analyses indicate HCC incidence trends have not declined uniformly across the U.S. census divisions. Despite ongoing public health efforts, disparities in liver cancer incidence and outcomes persist among Latinos [6,8]. These disparities may be influenced by a complex interplay of environmental, metabolic, behavioral, genetic, and social factors that remain underexplored in this specific geographic and demographic context; however, TX-based evidence related to these risk factors includes heterogeneous study designs with few studies evaluating multiple co-occurring risk factors in the same analytic models [31]. The present scoping review allows for a comprehensive mapping of the existing literature, identification of knowledge gaps, and informing future research.

### 1.3. Objectives of the Review

The objective of this scoping review is to identify, synthesize, and analyze existing literature on risk factors associated with HCC in Latino/a adults in Texas. Specifically, the authors of this review aim to map the range of risk factors evaluated in relation to this population, including alcohol use, aflatoxin exposure, chronic HBV and HCV, dietary patterns, genetic susceptibility, MetS, T2D, and obesity. Furthermore, this review seeks to identify gaps in the current evidence base to inform translational research directions and support the development of targeted prevention, intervention, and policy efforts aimed at reducing liver cancer disparities in Hispanic/Latino/a communities.

## 2. Materials and Methods

### 2.1. Research Team

For this scoping review, the research team included nutrition graduate research assistants (A.C., C.M.S., E.G., L.Y.T.K., S.G.) and a faculty member (R.S.P.), who specializes in nutrition and cancer research.

### 2.2. Procedures

Literature and Search Strategy. For this scoping review, the team followed guidance provided in the Preferred Reporting Items for Systematic Reviews and Meta-Analyses Extension for Scoping Reviews (PRISMA-ScR) to ensure comprehensive reporting of all stages of the review process [32] (Supplementary Materials). To identify relevant literature for this scoping review, peer-reviewed articles were retrieved using the PubMed and EbscoHost databases. Individual search strategies for each risk factor combined keywords related to the following core concepts: (1) Hispanic or Latino populations, (2) hepatocellular carcinoma (HCC), (3) Texas, and (4) specific risk factors, including aflatoxin, alcohol, diabetes, diet, genetics, MetS, and obesity. The literature search captured records as of 6 June 2025. The consistent search terms for PubMed included: [[“Hispanic or Latino” [MeSH Terms]] or [[American, Hispanic [MeSH Terms]] or [[“liver neoplasms” [MeSH Terms]] or [carcinoma, hepatocellular [MeSH Terms]] AND [[Texas [MeSH Terms] or [“Texas/epidemiology” [MeSH Terms]]. These terms were consistent across all searches, with variations only in the specific keywords related to the risk factors included in the search strategy. The consistent search terms for EbscoHost included: [liver cancer or hepatocellular carcinoma or liver neoplasms or metastatic liver cancer] AND [latino or hispanic or mexican or latina or latinos or latinas or latinx] AND Texas. Similar to PubMed, variations only occurred in the EbscoHost search strategy in the specific keywords related to the risk factors included in the search strategy. This scoping review addresses the following research question: What risk factors for HCC have been examined among Hispanic/Latino/a adults in TX across metabolic, environmental, viral, dietary, and genetic domains, and what gaps remain in the current body of literature?

### 2.3. Study Selection

**Figure 1:**
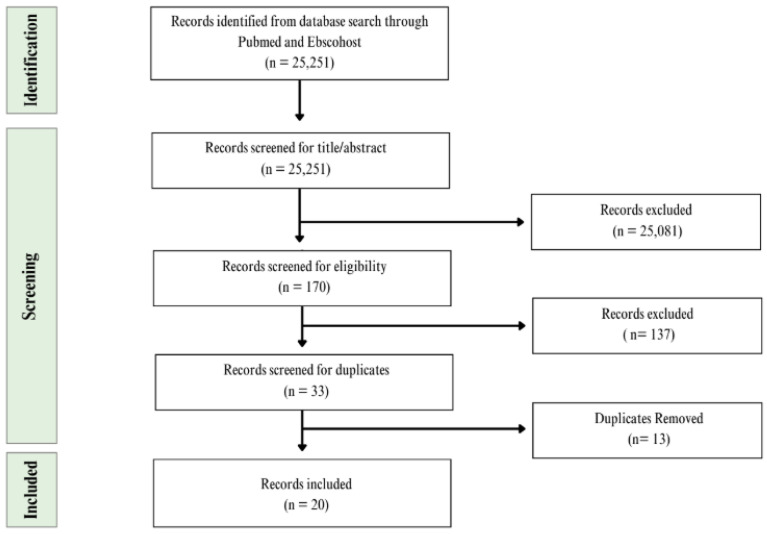
*Flow diagram of literature search.* Through a comprehensive search using Pubmed and Ebscohost databases, a total of 25,251 articles were identified. After the screening process, 170 articles were assessed for eligibility. Of these, 137 articles were excluded, and 20 articles were ultimately considered eligible and included in the scoping review analysis. The included studies comprised 9 articles related to metabolic-dysfunction-associated risk factors (obesity, T2D, and MetS) [4,6,16,31,33,34,35,36,37], 5 articles related to environmental exposures (aflatoxin and alcohol) [7,38,39,40,41], 2 articles examining infection- or virus-related exposures (HBV/HCV) [42,43], and 4 articles focused on genetic risk or predisposition [5,44,45,46]. Reasons for full-text exclusion included wrong population, wrong outcome, wrong exposure, non-original article type, and insufficient data to extract risk factor-specific outcomes.

The team conducted a three-phase screening process to select studies for inclusion. A primary screener developed the search strategy and conducted the initial screening of titles and abstracts to identify potentially relevant studies. A secondary screener then independently reviewed the same set of records using the same search strategy to confirm relevance. If the information in the title and abstract was insufficient to determine eligibility, the secondary screener reviewed the full text for alignment with PICOS criteria, where “P” stands for population, “I” is intervention or exposure, “C” is comparison, “O” is outcome, and “S” is study design as shown in Table 1. In the final phase, a third team member recorded eligible articles in a centralized tracking table used to organize and monitor study inclusion throughout the review process. Throughout all phases, checks and balances were maintained through ongoing discussion where any discrepancies or questions were brought to the lead investigator and the team for clarification. Studies were excluded if they lacked sufficient information to determine eligibility or if clarification could not be obtained. The screening process is documented in Figure 1, which depicts the number of records identified, screened, and included at each stage. This scoping review focused on the following risk factors for HCC among Latino adults in TX: aflatoxin, alcohol, diabetes, diet, genetics, HBV/HCV, MetS, and obesity. Inclusion criteria for this scoping review focused on Latino/Hispanic adults in TX, examining mentioned risk factors associated with HCC. Filters applied included: publication within the past 10 years, peer-reviewed articles, adults aged 18 years and older, and human studies. Included studies reported outcomes for Hispanic/Latino participants in Texas, either as 100% Hispanic cohorts (e.g., Cameron County Hispanic Cohort) or as mixed-ethnicity cohorts where Hispanic participants comprised a substantial subgroup with stratified or subgroup analyses. Studies that did not provide separate data for Hispanic/Latino participants or did not include Texas-based populations were excluded. Although only two included studies directly examined HBV/HCV-related outcomes, this should not be interpreted as evidence that viral hepatitis has a lesser impact as an HCC-related risk factor within Hispanic/Latino populations in south Texas. The included viral hepatitis studies assessed screening, linkage to care, and HCC surveillance implementation within safety-net settings, rather than estimating the full etiologic contribution of HBV or HCV to HCC incidence. Importantly, the nature of these screening studies suggests that viral hepatitis may be underdiagnosed in this population, as structured screening efforts identified hepatitis-related infection and cirrhosis among patients who may otherwise have faced barriers to diagnosis, referral, and follow-up care. Therefore, the limited number of HBV/HCV-focused studies may reflect gaps in screening access and documentation, rather than reduced risk factor importance. The studies that included genetic risk are limited by sample size and study design. Several genetic and molecular studies included are limited by their small sample sizes, using small numbers of Hispanic HCC cases, selected tumor or serum specimens, or including subgroup analyses within broader non-Hispanic cohorts. Due to this, the statistical power and ability to detect genetic associations and evaluate heterogeneity across Latino subgroups are unknown.

### 2.4. Data Extraction of Included Studies

Four reviewers (R.S.P., L.Y.T.K., E.G., and C.M.S.) extracted data from the included studies into Word tables. Information was organized in tables developed for data extraction. The reviewers extracted general study information as shown in Table 2, including the following variables: author, year, article title, risk factor, location, study design, age, mean age, total population, gender distribution, sample size, race/ethnicity, city or county, intervention status, and intervention details. Once study selection and data extraction were completed, additional tables were created to categorize outcomes by specific risk factors: Table 3 (RSP and CMS), outcomes related to diabetes, MetS, and obesity; Table 4 (LYTK), outcomes related to aflatoxin and alcohol; Table 5 (EG), outcomes related to hepatitis B and hepatitis C; and Table 6 (EG), outcomes related to genetics.

Given the heterogeneity in study designs and outcomes, we did not attempt a formal meta-analysis. Instead, we used a narrative and tabular approach to compare the direction and magnitude of associations within each risk factor domain (metabolic, environmental, viral, genetic). When findings were conflicting across studies (e.g., differences in effect size estimates or non-overlapping confidence intervals), we prioritized interpretation based on study quality indicators (sample size, study design, and adjustment for key confounders) and consistency of results within the same risk factor category, while explicitly highlighting remaining uncertainty in the text and tables. When studies included mixed-ethnicity cohorts, we extracted effect estimates that were specific to Hispanic/Latino participants whenever reported. For studies that only provided overall estimates in mixed populations but with a substantial proportion of Hispanic participants, we used these findings descriptively to contextualize risk but did not treat them as strictly population-specific estimates.

Reviewers also documented comments and clarifications in a separate, unpublished notes column to support consistency and transparency during data review. Each reviewer was assigned a set of studies for extraction and cross-reviewed other sections for accuracy. Any issues, discrepancies, or questions were discussed collaboratively amongst the team to ensure data accuracy and consensus.

## 3. Results

### 3.1. Overview

**Figure 2:**
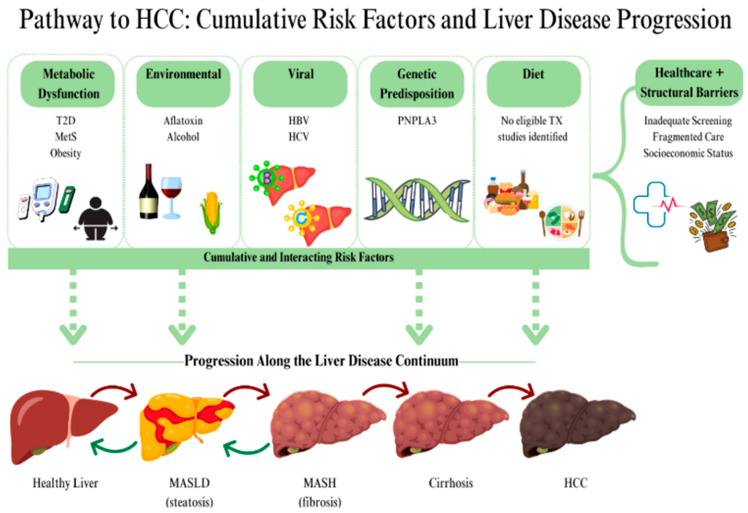
*Pathway to HCC: Cumulative Risk Factors and Liver Disease Progression.* This image illustrates how cumulative and interacting risk factors contribute to progression along the liver disease continuum culminating in HCC amongst Hispanic/Latino/a populations in Texas. Risk factors represented include metabolic dysfunction (type 2 diabetes (T2D), metabolic syndrome (MetS), obesity), environmental exposures (alcohol, aflatoxin), viral hepatitis (hepatitis B virus (HBV), hepatitis C virus (HCV), genetic predisposition (PNPLA3), dietary factors (for which no eligible Texas-based studies were identified in this review), and healthcare and structural barriers (including inadequate screening, fragmented care, and socioeconomic disadvantages). These risk factors are shown as converging and interacting upstream determinants that collectively influence disease progression from a healthy liver to MASLD, metabolic-dysfunction-associated steatohepatitis (MASH), cirrhosis, and HCC. Red arrows depict forward disease progression, while green arrows indicate stages at which liver injury may be partially reversible with risk factor modification or intervention. Dashed arrows indicate that risk factors may act at multiple stages of disease rather than in a linear manner alone. The figure synthesizes evidence from the present scoping review and indicates points where cumulative risk may accelerate progression along the liver disease continuum amongst Hispanic/Latino/a populations in Texas.

Figure 1 outlines the screening and selection process. The initial search for all included risk factors yielded 25,251 results (n = 25,251) across PubMed and Ebscohost databases. After title and abstract screening, this was narrowed to 170 articles (n = 170). Of those, 33 articles (n = 33) met the inclusion criteria. Duplicates were then removed; duplicates also included overlapping articles between databases and articles appearing in multiple risk factor searches, as some studies addressed more than one outcome of interest. Following this process, 20 articles (n = 20) were included in the final scoping review as shown in Table 2 (CMS, LYTK, EG). The included studies comprised nine articles related to metabolic-dysfunction-associated risk factors (obesity, T2D, and MetS), five articles related to environmental exposures (aflatoxin and alcohol), two articles examining infection- or virus-related exposures (HBV/HCV), and four articles focused on genetic risk or predisposition. As seen in Table 2, the 20 included articles in this scoping review include: Das et al. (2024) [5]; El-Serag et al. (2021) [6]; Garza et al. (2016) [33]; Gill et al. (2017) [38]; Gudenkauf et al. (2020) [35]; Hatia et al. (2025) [31]; Jiao et al. (2016) [34]; Jiao et al. (2018) [7]; Jiao et al. (2021) [41]; Lee et al. (2021) [4]; Lopez et al. (2024) [39]; Ma et al. (2022) [46]; Pollock et al. (2016) [42]; Ramirez et al. (2017) [45]; Sharpton et al. (2023) [44]; Singal et al. (2017) [37]; Taylor et al. (2016) [40]; Thrift et al. (2023) [43]; Thrift et al. (2024) [36]; and Turner et al. (2019) [16]. Figure 2 details the risk factors examined across included studies evaluating HCC among Hispanic/Latino/a populations in Texas in the present scoping review. For interpretability, we grouped risk factors into four primary domains: metabolic (T2D, obesity, MetS), environmental (alcohol, aflatoxin), viral (HBV, HCV), and genetic (tumor somatic mutations, germline variants, polygenic risk scores). These domains structured both our data extraction (Table 3, Table 4, Table 5 and Table 6) and the narrative synthesis.

### 3.2. Study Design and Intervention Foci

As shown in Table 2, of the twenty included studies included in this scoping review, the majority (n = 11) were observational in design [4,5,11,16,31,33,34,35,38,39,44]. Three studies were prospective, one cohort, one cross-sectional, one multivariate, one matched case–control, one translational case–control and in vitro investigation, and one double-blind RCT. Several studies relied on established cohorts and population-based databases to identify participants, capture longitudinal clinical data, or link outcomes related to HCC risk. Six studies utilized the Cameron County Hispanic Cohort (CCHC) as their primary database [7,33,34,35,36,44]. Two included studies utilized statewide cancer registry data through the Texas Cancer Registry (TCR) and U.S. National Program of Cancer Registries and Surveillance, Epidemiology, and End Results Program (SEER) to examine HCC trends alongside risk factor contributions [12,38]. Three additional studies incorporated additional large clinical cohorts or databases for analysis including the Hispanic Liver Cancer Cohort (HLCC), Texas Hepatocellular Carcinoma Consortium Cohort (THCCC), and Houston Veterans Administration Cirrhosis Surveillance Cohort (HVASC) [36,39,45]. The remaining studies did not reference the use of the aforementioned databases or resources were not applicable to their study design [4,5,16,40,42].

Across the included studies included in this scoping review, two incorporated an active intervention component: Lopez et al. (2024) and Pollock et al. (2016) [39,42]. Lopez et al. implemented a behavioral intervention through *Salud y Vida*, which focused on T2D management through health coaching and diabetes self-management education [37]. In contrast, Pollock et al. conducted a double-blind, placebo-RCT evaluating ACCS100 in low- and high-dosing strategies to reduce aflatoxin exposure among predominantly Hispanic/Latino adults in south TX [41]. While both culturally relevant interventions targeted south TX communities, they aimed to address different risk factors: one targeting environmental toxin reduction (aflatoxin) [41] and the other targeting metabolic disease management (T2D) [37].

### 3.3. Participant Characteristics

Regarding age criteria, seven studies enrolled adults ≥ 18 years old [31,33,35,36,37,40,41], three studies included adults ≥ 25 years old [11,34,38], and five studies focused on older adult populations ≥ 49.5 years old [16,39,42,44,45]. Mean age reporting varied across studies. While the majority of studies (n = 13) reported a mean age ranging between 45.2 and 63 years old [7,15,16,31,33,34,35,36,37,39,42,44,45], several studies (n = 6) did not report mean age [5,6,37,38,40]. Das et al. included the smallest sample size (n = 109, HCC tumor tissue samples) [5] whereas Lee et al. reported the largest cohort (n = 299,116) [4]. Across all included studies, the total combined sample size was 424,749 participants [5,7,11,15,31,33,34,35,36,37,41,42,44,45]. Additionally, gender data were reported in most studies, with a total of 10,516 females (F) and 11,327 males (M) across those providing sex distributions. Notably, three studies did not report F/M distribution.

### 3.4. Setting

**Figure 3:**
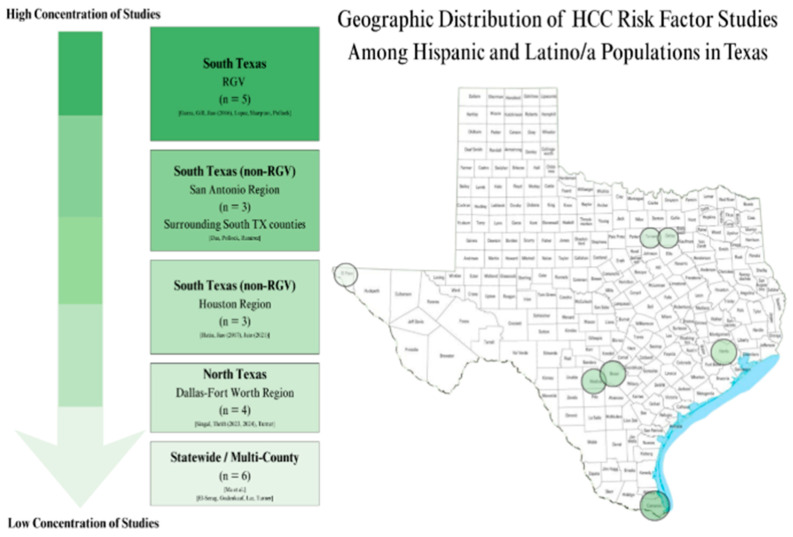
*Geographic Distribution of HCC Risk Factor Studies Among Hispanic/Latino/a Populations in Texas.* Included studies are mapped by primary study setting and grouped by geographic region, particularly the Rio Grande Valley (RGV), as well as representation in the San Antonio, Houston, and Dallas–Fort Worth regions. Of the included studies, 5 studies were conducted in the RGV, 3 in the San Antonio region, 3 in the Houston region, 4 in north Texas, and 6 utilized statewide or multicounty Texas samples. Shading intensity reflects the relative concentration of studies by region, with darker shading indicating higher study density. This figure illustrates the strong regional focus of existing evidence in south Texas Hispanic/Latino/a populations.

Geographic distribution of HCC risk factor studies among Hispanic/Latino/a populations in Texas included in this systematic review can be found in Figure 3. Most of the articles included in this scoping review (n = 11) were based in south TX, with five articles specific to the Rio Grande Valley (RGV) [5,7,31,33,34,35,36,37,40,41,44]. All remaining articles were TX based. Study settings spanned several Texas cities, including Brownsville, Dallas, El Paso, Fort Worth, Galveston, Harlingen, Houston, McAllen, and San Antonio [5,15,31,33,34,35,36,37,39,42,45]. For studies that specified county-level detail, these locations corresponded to regions such as Cameron County, Dallas County, Harris County, Medina County, Bexar County and its seven surrounding counties, and multicounty areas in the Houston region [7,40,41]. Additional geographically broad studies encompassed statewide samples, six health systems across north and south Texas, and 32 counties located within 100 miles of the U.S. and Mexico border [11,16,38]. The RGV, particularly Brownsville and the surrounding Cameron County region, was the most frequently represented geographic area in the included literature, with five studies conducted in this setting. Collectively, these studies represented 9415 participants, indicating strong regional representation despite this total being substantially smaller than the largest single study in the review [4,33,34,35,36,37].

### 3.5. Outcomes Related to T2D, MetS, and Obesity

Table 3 summarizes study characteristics examining metabolic-dysfunction-related risk factors, including T2D, MetS, and obesity and their associations with liver disease progression and HCC risk [4,6,16,31,33,34,35,36,37]. Across studies included in Table 3, measurable outcomes cluster around population-level incidence, early metabolic liver disease, advanced fibrosis and cirrhosis, and HCC risk and prognosis, with metabolic dysfunction examined across disease stages and progression [4,6,16,31,33,35,36,37,44]. As defined by the inclusion criteria, evidence summarized mainly derives from south TX and the RGV, particularly Cameron County, allowing for consistent evaluation of metabolic dysfunction within a geographically and demographically focused Mexican American population.

#### 3.5.1. Population Burden

Several studies (n = 2) analyzed population-level HCC incidence rates across regions and demographic groups and found consistently higher HCC incidence rates in TX, amongst Hispanic/Latino populations, with increasing trends over time [4,6]. Population-based registries were utilized to measure age-adjusted HCC incidence rates across U.S. states and regions, finding incidence rates in TX were consistently higher than national rates across sex, race, ethnicity, and age group when compared to national averages. According to El Serag et al., across populations, males had approximately threefold higher HCC incidence than females, although increased trends were observed in both sexes [6]. HCC incidence was highest amongst middle-aged and older adults (55–74 years), with elevated rates observed in south TX and U.S.–Mexico border regions compared with the rest of TX [6].

#### 3.5.2. Early Markers/Liver Disease

Several cohort studies (n = 2) measured the prevalence of early metabolic liver disease using imaging-based assessments and found high burden of NAFLD, steatosis, and fibrosis among Mex. Americans, including younger adults and first-degree relatives of HCC patients. These studies reported high prevalence of NAFLD, hepatic steatosis, and significant fibrosis, with metabolic factors such as obesity and T2D frequently associated with disease presence and progression [33]. In one cohort, NAFLD assessed via liver ultrasound was highly prevalent (~49%) and was independently associated with subclinical disease markers in younger participants, including increased carotid intima media thickness among adults <45 years [33]. According to Sharpton et al., while examining first-degree relatives of Mex. Americans with HCC, 42% had definite hepatic steatosis and 17% had significant fibrosis, with fibrosis prevalence increased to 20% among individuals ≥ 40 years and 5% meeting criteria for suspected cirrhosis [36]. Across both studies, early liver disease and fibrosis were frequently observed in individuals with metabolic dysfunction, with T2D and obesity commonly associated with disease presence [33,36].

#### 3.5.3. Progression of Liver Disease (Fibrosis and Cirrhosis)

Building on findings of high steatosis and early fibrosis prevalence associated with metabolic dysfunction results previously reported, other studies (n = 2) evaluated liver disease using non-invasive fibrosis indices (APRI or FIB 4) and found elevated prevalence of fibrosis and cirrhosis amongst Hispanic/Latino populations in south TX, particularly prevalent amongst individuals with diabetes, obesity, or central adiposity risk factors [16,34]. In a community-based cohort, cirrhosis and advanced fibrosis prevalence exceeded national estimates with the highest prevalence observed among males, including younger adults aged 25–34 years, and with diabetes and central obesity identified as key risk factors [34]. Notably, in addition to metabolic risk factors, PNPLA3 risk alleles were associated with higher APRI scores and increased odds of cirrhosis and advanced fibrosis, particularly among older participants [34]. According to Turner et al., utilizing an EMR-based analysis of patients at the time of HCV diagnosis, advanced liver disease was present in nearly one quarter of patients, with Hispanic/Latinos demonstrating higher odds compared with non-Hispanic White and Black populations [16]. Notably, although Turner et al. included insurance as a covariate to account for access to care and disease severity at presentation, it was not independently associated with advanced liver disease, and adjustment for insurance did not attenuate higher odds of advanced liver disease observed amongst Hispanic/Latino with diabetes and obesity, demonstrating observed disparities were not driven by differences in access to care alone [16]. Across both studies, metabolic dysfunction, particularly T2D and obesity, remained consistently associated with increased progression and advancement of liver disease, with alcohol use and older age further contributing to disease severity [16,34].

#### 3.5.4. Metabolic Dysfunction Risk Factors Influence HCC Risk and Outcomes

Consistent with previous findings associating metabolic dysfunction with fibrosis and cirrhosis development and progression amongst Mex. Americans, several studies (n = 2) found that the same risk factors of T2D, MetS, and obesity were associated with increased cancer risk, including higher HCC risk and altered prognosis [31,35]. These studies reported diabetes as a strong risk factor for cancer and HCC, with longer diabetes duration associated with higher risk and metformin use associated with improved survival. In a large RGV community-based cohort, diabetes was identified as the strongest risk factor of cancer among participants under age 70, with liver cancers ranking high and higher frequency observed among first- and second-degree relatives. Local age-adjusted data further confirmed elevated liver cancer incidence among Cameron County Hispanics compared with non-Hispanic populations [35]. In a separate case–control analysis, T2D was independently associated with increased HCC risk, with longer diabetes duration demonstrating a dose-dependent relationship [31]. Additionally, this study also reported combined effects between diabetes, viral hepatitis infection, and heavy alcohol use on HCC risk, while metformin use was associated with improved survival amongst HCC patients with T2D [31].

#### 3.5.5. Targeted Care/Intervention

Given the high burden of metabolic dysfunction and its consistent association across liver disease stages and the development and progression of HCC in south TX, one intervention study (n = 1) evaluated a community-health-worker-integrated chronic care model and measured changes in blood pressure, as a measurable marker of cardiometabolic control reflecting the management of multiple chronic conditions, reporting sustained improvements among Hispanics with co-occurring T2D and HTN [37]. Amongst 3806 adults with poorly controlled cardiometabolic disease, significant reductions in systolic and diastolic blood pressure were observed within three months and were sustained for up to 24 months [37]. Participants with higher program engagement experienced greater and more sustained reductions in systolic blood pressure compared with lower engagement groups. The study population was characterized by multiple chronic conditions and reported fragmented care, highlighting the relevance of a targeted care delivery approach within metabolically high-risk south TX populations [37].

### 3.6. Outcomes Related to Aflatoxin and Alcohol

Table 4 summarizes studies examining environmental exposure-related risk factors, including alcohol consumption and aflatoxin exposure and their associations with HCC risk among Latino populations in Texas [7,38,39,40,41]. Across studies included in Table 4, measurable outcomes cluster around population-level cancer burden attributable to alcohol, biomarkers of aflatoxin exposure, molecular signatures of aflatoxin-associated mutations, and HCC case–control comparisons evaluating exposure prevalence. As defined by the inclusion criteria, evidence summarized derives primarily from south Texas and the Rio Grande Valley, with studies utilizing population-based cancer registries, community-based cohorts, and clinical case–control designs, allowing for consistent evaluation of environmental exposures within Hispanic/Latino populations.

#### 3.6.1. Alcohol Consumption and HCC Burden

Two studies examined the contribution of alcohol consumption to cancer burden and evaluated the effect of synergistic interactions between alcohol use and genetic predisposition on HCC risk [38,39]. At the population level, Gudenkauf et al. (2020) [35] analyzed Texas Cancer Registry data and found that 2.9% of all cancers diagnosed in Texas in 2015 were attributable to alcohol consumption, with a slightly elevated burden among Hispanic/Latino populations (3.0%) compared to non-Hispanic Whites (2.7%) and non-Hispanic Blacks (2.2%), with men demonstrating higher attributable fractions (3.6%) than women (2.2%). In addition to population-level contributions, Thrift et al. (2024) [36] examined the effect of synergistic interactions between heavy alcohol consumption and genetic variants on HCC risk among cirrhosis patients, demonstrating that heavy alcohol use exerts multiplicative effects with genetic predisposition. Specifically, among a prospective cohort of 1911 cirrhosis patients (28.7% Hispanic), cirrhosis patients carrying the PNPLA3 G risk allele who engaged in current heavy alcohol consumption had 2.65-fold higher HCC risk (HR 2.65, 95% CI: 1.20–5.86, *p* < 0.05) compared to non-carriers without heavy drinking [39]. These findings underscore that alcohol consumption contributes substantially to HCC burden among Hispanic/Latino populations, and genetic susceptibility may modify individual risk trajectories.

#### 3.6.2. Aflatoxin Exposure and Molecular Evidence of Exposure

A few other studies (n = 2) provided evidence of ongoing aflatoxin exposure among Hispanic/Latino populations in Texas, utilizing both molecular biomarkers and direct detection of aflatoxin in biological specimens [7,40]. Jiao et al. (2018) [7] identified the TP53R249S mutation, a molecular hallmark of aflatoxin exposure, in tumor and plasma samples from Hispanic HCC patients in south Texas. Among 41 Hispanic HCC tumor samples analyzed using droplet digital PCR and restriction fragment length polymorphism, the TP53R249S mutation was detected in 7.3% (3/41) of tumors and in 5.7% of plasma cell-free DNA samples from 218 HCC patients seeking care at a major cancer center [7]. Notably, patients harboring the TP53R249S mutation were significantly younger (mean age 55.7 years) compared to those without the mutation (mean age 64.1 years) and demonstrated shorter overall survival, with the mutation detected exclusively in Hispanic and Asian patients and never observed in non-Hispanic populations [7].

Building on molecular evidence, Ramirez et al. (2017) [45] conducted a case–control study comparing 51 HCC cases and 104 matched controls in south Texas (67% Latino; 72.5% of cases male) and provided direct evidence of contemporary aflatoxin exposure by measuring aflatoxin biomarkers in blood and urine specimens. HCC cases demonstrated significantly higher odds of detectable aflatoxin biomarkers in serum (OR 6.09, 95% CI: 1.10–33.71) and urine (OR 3.42, 95% CI: 1.07–10.91) compared to matched controls, indicating substantially elevated aflatoxin exposure among individuals with HCC. Importantly, HCC cases in this study were also significantly more likely to have Medicare or Medicaid insurance, lower income, and less education than controls and demonstrated higher lifetime alcohol and tobacco use. Cases showed markedly elevated odds of hepatitis C infection (OR 183.74, 95% CI: 27.37–∞) and cirrhosis (OR 2.17, 95% CI: 33.3–∞), with cases less likely to be taking potentially protective medications including aspirin (OR 0.31, 95% CI: 0.11–0.85), statins (OR 0.03, 95% CI: 0–0.20), or omega-3/fish oil supplements (OR 0.10, 95% CI: 0.01–0.78). Across both studies, evidence of aflatoxin exposure, whether detected as molecular signatures in tumor DNA or as biomarkers in biological specimens, was consistently elevated among Hispanic/Latino HCC patients, with exposure associated with younger age at diagnosis and more aggressive disease phenotypes.

#### 3.6.3. Intervention to Reduce Aflatoxin Bioavailability

Given the documented burden of aflatoxin exposure in south Texas Hispanic/Latino populations, one randomized controlled trial evaluated an intervention strategy to reduce dietary aflatoxin bioavailability [41]. Pollock et al. (2016) [42] enrolled 234 participants (100% Hispanic; 180 females, 54 males; age range 18–77 years) from Bexar and Medina Counties in a double-blind, placebo-controlled trial of calcium montmorillonite clay (ACCS100) administered over three months. The low-dose ACCS100 group (1.5 g/day) demonstrated a significant reduction in serum AFB_1_-lysine adduct levels by month 3 (*p* = 0.0005), indicating reduced dietary aflatoxin bioavailability. Notably, participants in the study region consumed corn tortillas significantly more frequently than the national average (56% vs. 20% consuming daily), highlighting a prevalent dietary exposure route in this population. The study reported no significant changes in serum biochemistry or hematology across treatment groups, suggesting the safety of the intervention. This intervention trial demonstrates a practical and feasible strategy to mitigate aflatoxin exposure and biomarker detection in this population with documented chronic exposure to this hepatocarcinogen.

#### 3.6.4. Synthesis of Environmental Exposures and HCC Risk

Collectively, evidence from Table 4 demonstrates that environmental exposures, specifically alcohol consumption and aflatoxin contamination, substantially contribute to HCC risk among Hispanic/Latino populations in Texas. Alcohol consumption accounts for 3.0% of cancer cases among Hispanics and exerts synergistic effects with genetic predisposition (PNPLA3 variants), amplifying HCC risk among cirrhosis patients. Aflatoxin exposure is documented through multiple pathways: as molecular signatures (TP53R249S mutations) in tumor DNA, as circulating biomarkers (AFB_1_-lysine adducts) in blood and urine, and through biogeographic trends suggesting increasing exposure over time in some Texas regions [7,38,39,40,41,45]. Social determinants of health, including lower income, reduced healthcare access, and lower medication utilization, appear intertwined with higher exposure burden, particularly evident in the Ramirez et al. study where HCC cases showed elevated aflatoxin biomarkers alongside markers of healthcare disadvantage. The availability of a safe, low-cost intervention (calcium montmorillonite clay) that significantly reduces aflatoxin bioavailability offers promise for population-level HCC prevention strategies in high-risk communities [41]. Together, these findings underscore that modifiable environmental exposures and their synergistic interactions with genetic susceptibility represent critical leverage points for HCC prevention and health equity initiatives among Texas Hispanic/Latino populations.

### 3.7. Outcomes Related to Hepatitis B and Hepatitis C

Table 5 summarizes the study characteristics examining virus-related exposures, specifically hepatitis B (HBV) and hepatitis C (HCV), within the context of liver disease and HCC risk [42,43]. Across studies included in Table 5, measurable outcomes cluster around implementation of various screening strategies at different stages of the liver-disease-to-HCC continuum, including (1) identification of HCV infection exposure and current active infection in hospital-based populations [42] and (2) participation in HCC surveillance among patients with established cirrhosis [43]. Evidence summarized reflects a period in which viral hepatitis represented the predominant etiologic driver of HCC and focuses on screening delivery within large Texas safety-net systems serving substantial Hispanic/Latino populations. As defined by the inclusion criteria, evidence summarized is derived primarily from the south Texas region, with studies using intervention-focused designs as well as population- and community-based registries to evaluate hepatitis-related exposure, HCC risk, and screening intervention effectiveness within a predominantly Hispanic/Latino population.

#### 3.7.1. Hepatitis C Screening and Infection Burden at Early Stages of Liver Disease

One hospital-based implementation study (n = 1) evaluated outcomes of hepatitis C virus (HCV) screening among baby boomer patients (1945–1965) receiving care at a south Texas safety-net hospital for 10 months between 2013 and 2014 [42]. Screening outcomes included testing for anti-HCV antibody positivity, confirming prior exposure, and HCV RNA positivity, indicating current active infection. Among 2327 screened patients, 192 (8%) were anti-HCV positive, of whom 107 (56%) were Hispanic/Latino [42]. These patients represent nearly four times the prevalence reported among Hispanic individuals of similar Mexican descent in national and community-based cohorts, including the National Health and Nutrition Examination Surveys (NHANESs) 2007–2010, and the Hispanic Community Health Study/Study of Latinos (HCHS/SOL). These patients were found more likely to be male (OR 3.02, 95% CI: 2.13, 4.30) and younger (56 vs. 58 years, respectively, *p* < 0.001) [42]. One hundred and sixty-seven of those patients with anti-HCV positivity were tested for presence of active HCV infection; a substantial portion demonstrated current active HCV infection based on RNA testing (65%) [42]. Hispanic/Latino patients of this active HCV cohort were found to be younger (mean age 54 vs. 56 years, respectively, *p* = 0.029) and more likely to be male (OR 3.71, 95% CI: 1.31, 10.50) [42]. Measured outcomes across the screening and recruitment period also included post-test counseling, referral, specialty care attendance and treatment initiation. Counseling was documented in 88% of patients, 77% were referred to primary care, 56% were referred to specialty care, 34% attended a specialty appointment, and fewer than 20% initiated antiviral treatment [42]. Reported barriers among patients with chronic HCV included lack of insurance, substance abuse, incarceration history, and unstable housing. These barriers were found to be more common in newly diagnosed patients who self-identified as Hispanic vs. non-Hispanic, with 57% of chronically infected Hispanic/Latino patients reporting lack of health insurance [42]. These findings document the burden of hepatitis C exposure and active infection as well as potential underlying causes in barriers to care, identified through hospital-based screening in a majority Hispanic/Latino south Texas cohort during this study period.

#### 3.7.2. HCC Surveillance Implementation Among Patients with Advanced Liver Disease

One prospective, pragmatic randomized controlled implementation study (n = 1) aimed to increase ultrasound-based HCC screening participation among patients with cirrhosis within a large safety-net hospital system in Dallas, Texas [43]. Singal et al. evaluated differences in HCC surveillance and patient recruitment when comparing usual care, mailed outreach alone, and mailed outreach plus patient navigation support. The study was conducted from December 2014 through March 2016. Measurable outcomes included one-time HCC screening participation and time-to-response following outreach invitations. At the conclusion of the trial, Singal et al. found that mailed outreach doubled HCC screening rates compared with usual care in this safety-net health system. Hepatitis-related infection represented the predominant etiology of liver disease, with HCV accounting for 51% and HBV for 3.4% of cases among recruited patients across all treatment arms [43]. Hispanic/Latino patients comprised 37.8% of the patient populations, representing the predominant racial group across treatment arms [43]. When looking at characteristics within this recruited patient population, Hispanic/Latino patients were found to be more likely to participate in HCC screening compared with non-Hispanic White patients (AOR 1.56, 95% CI: 1.20–2.02) [43]. Male sex was associated with lower screening participation (AOR 0.80, 95% CI: 0.65–0.99), despite representing a target demographic for HCC surveillance [43]. Increased age was associated with modestly higher screening participation (OR 1.52, 95% CI: 1.20–1.93) [43]. Engagement with primary care (AOR 1.05, 95% CI: 1.03–1.08) and gastroenterology specialist visits (AOR 1.74, 95% CI: 1.35–2.21) was also identified as a result of increased screening participation [43]. Outreach-based recruitment strategies were associated with decreased time-to-response compared with usual care. Collectively, these findings highlight the predominance of hepatitis-related cirrhosis within this safety-net population and document differences in HCC screening participation across treatment strategies and patient subgroups.

### 3.8. Outcomes Related to Genetics

Table 6 summarizes study characteristics examining genetic risk and genetic predisposition, as well as molecular features associated with HCC among Hispanic/Latino populations [5,44,45,46]. Across studies in Table 6, measurable outcomes cluster around (1) tumor-specific somatic mutation frequencies and pathway alterations, (2) non-invasive molecular detection and risk profiling using circulating biomarkers, and (3) germline genetic risk stratification for incident HCC among patients with cirrhosis [5,39,44,46]. As defined by the inclusion criteria, evidence summarized is derived primarily from south-Texas-based cohorts and large U.S. multiethnic datasets, enabling evaluation of genetic and molecular features relevant to Hispanic/Latino populations. Most genetic studies included in this review were conducted among Mexican American adults in south Texas or within multiethnic U.S. cirrhosis cohorts in which Hispanics were predominantly of Mexican origin. These populations are characteristically admixed, with substantial Indigenous American and European ancestry and lower but non-trivial African ancestry. As a result, reported PNPLA3 allele frequencies and performance of hepatic fat polygenic risk scores likely reflect this specific admixture profile and may not directly generalize to Hispanic subgroups with different ancestry proportions, such as Caribbean origin populations.

#### 3.8.1. HCC Tumor Multiomics Characterization in South Texas Cohort

One integrative multiomics study (n = 1) evaluated molecular alterations in HCC tumors from a south Texas Hispanic cohort using paired tumor and adjacent non-tumor liver tissue. Whole-exome sequencing, TERT promoter sequencing, RNA sequencing, proteomic mass spectrometry, metabolomic profiling, and serum lipidomic analyses were performed and compared with other known HCC cohorts, such as the Liver Hepatocellular Carcinoma (LIHC) cohort in The Cancer Genome Atlas (TCGA), as well as other international cancer genomics cohorts. Across 27 tumor samples, exome sequencing identified 1528 somatic variants across 27 tumors with a median non-silent mutation burden of 1.06 mutations/Mb (range 0.04–2.28) [5]. *AXIN2* mutations were detected in 11.1% of tumors in the Hispanic cohort, occurring at significantly higher frequencies compared to non-Hispanic White patients in TCGA-LIHC (11.1% vs. 0.6%; *p* = 0.00912) [5]. Compared with African American patients in TCGA-LIHC, the south Texas Hispanic cohort demonstrated a lower *TP53* mutation frequency and a higher *CTNNB1* mutation frequency (*p* = 0.00032) [5]. TERT promoter mutations, primarily CC28T, were identified in 77.8% of south Texas Hispanic tumors, exceeding frequencies reported in TCGA-LIHC White (47.8%, *p* = 0.00535) and Asian (31.5%; *p* = 0.00012) cohorts [5]. Transcriptomic, proteomic, and metabolomic analyses further characterized pathway-level alterations involving Wnt/β-catenin signaling and lipid metabolism within this cohort [5].

#### 3.8.2. cfDNA Somatic Mutation Profiling for HCC Risk and Prognosis

One case-comparison study (n = 1) evaluated somatic mutations in circulating cell-free DNA (cfDNA) among Hispanic/Latino patients with HCC compared with Hispanic/Latino patients with advanced liver fibrosis but no HCC [44]. Plasma samples were analyzed using targeted sequencing of cancer-associated genes, and clinical variables including APRI score, family history of HCC, type 2 diabetes, and alcohol consumption were recorded [44]. Among Hispanic patients with HCC, non-synonymous somatic mutations were detected in 22 of 27 participants, with *TP53* identified as the most commonly mutated gene (27%) followed by *NFE2L2* and *CTNNB1* (14% each) and *KMT2D*, *AXIN1*, *AR*, and *BIVM-ERCC5* (9% each) [44]. Higher cfDNA concentrations and increased mutation counts were observed among patients with HCC and were associated with shorter overall survival (*p* < 0.001 and *p* = 0.045, respectively) [44]. In the advanced fibrosis group without HCC, non-synonymous somatic mutations were identified in 17 of 51 participants, with *KMT2D* reported as the most frequently mutated gene [44]. Somatic mutations in *KMT2D* were correlated with advanced fibrosis and cirrhosis (17.6%) in this group [44].

#### 3.8.3. Serologic Autoantibody Biomarkers for HCC Detection in Hispanic Cohort

One biomarker discovery and validation study (n = 1) investigated tumor-associated antigen (TAA) autoantibodies as diagnostic markers for HCC among Hispanic/Latino patients [46]. Candidate TAAs were identified using serologic proteome analysis (SERPA) and differential gene expression analysis, followed by ELISA-based validation in serum samples from Hispanic/Latino patients with HCC (n = 224), cirrhosis (n = 20), chronic hepatitis (n = 26), and healthy controls (n = 40). Autoantibodies targeting DNMT3A (45.8%), p16 (41.7%), HSP60 (37.5%), and HSPA5 (33.3%) were significantly elevated in Hispanic/Latino HCC sera compared with non-cancer controls [46]. Individually, these autoantibodies demonstrated receiver operating characteristic (ROC) area-under-the-curve (AUC) values ranging from 0.7505 to 0.8885 [46]. When combined into a four-marker panel, overall sensitivity for HCC detection increased to 75%, compared with the highest sensitivity observed for any single autoantibody (45.8%) [46].

#### 3.8.4. Germline Polygenic Risk Stratification for HCC Among Cirrhosis Patients

One prospective cohort study (n = 1) evaluated a hepatic polygenic risk score (PRS), incorporating germline variants in PNPLA2, TM6SF2, MBOAT7, and GCKR, for predicting incident HCC among patients with cirrhosis enrolled across multiple U.S. health systems [45]. Clinical data including anthropometrics, diabetes, dyslipidemia, hypertension, alcohol use, and viral hepatitis status were recorded. HCC risk increased by 134% per unit increase in PRS (HR 2.30; 95% CI: 1.35–3.92) [45]. Patients in the highest PRS tertile demonstrated approximately two-fold higher HCC risk compared with those in the lowest tertile (HR 2.05; 95% CI: 1.22–3.44) [45]. The frequency of the PNPLA3 G allele was highest among Hispanic/Latino participants (65%) [45]. The cohort included multiple etiologies, including 42.1% heavy alcohol use and 19.5% active HCV infection [45].

## 4. Discussion

The present scoping review synthesized and analyzed 20 research studies published through 6 June 2025 examining HCC risk factors among Hispanic/Latino adults in TX, with evidence most frequently addressing metabolic dysfunction and less frequently addressing viral hepatitis and genetics; notably, studies directly examining dietary exposures or diet-related patterns in relation to HCC risk in this population were unable to be identified [4,5,6,7,16,31,33,34,35,36,37,38,39,40,41,42,43,44,45,46]. To our understanding, this review represents the most comprehensive synthesis and review of TX-based evidence on HCC risk amongst Hispanic/Latino/a populations across metabolic, environmental, viral, genetic, and intervention domains. Findings from this review directly relate to researchers, clinicians, and public health practitioners seeking to better understand HCC etiology and inform prevention, screening, and intervention strategies tailored to high-risk Hispanic/Latino/a communities in TX. This discussion synthesizes findings across metabolic, environmental, viral, and genetic domains to contextualize how overlapping risk factors contribute to HCC development among Hispanic/Latino/a populations in TX, with each theme examined from a distinct etiologic and translational perspective.

### 4.1. Metabolic Dysfunction and Risk Factor Interactions Across Liver Disease Stages

Metabolic dysfunction was consistently associated with liver disease burden across pathology stages, from early steatosis and fibrosis to cirrhosis and HCC risk and prognosis, within predominantly south TX Mexican American populations [4,16,31,33,34,35,36]. While this pattern suggests that diabetes, obesity, and MetS represent recurring commonalities/issues/overlapping (or something) across multiple stages of disease, the evidence presented remains limited in its ability to define how these metabolic exposures interact with other established HCC risk factors, including alcohol consumption, viral hepatitis, genetics, and diet considerations within Mexican American, Hispanic, and Latino/a populations [16,31,34]. Importantly, only select studies evaluated metabolic dysfunction alongside additional co-occurring risk factors within the same analytic models, including alcohol and hepatitis exposures [16,31,34]. For example, Hatia et al. reported synergistic effects between T2D, alcohol use, and viral hepatitis infection on HCC risk [31]. However, collectively, few studies directly evaluated multirisk factor exposures to HCC outcomes in TX Latino/as, limiting clarity regarding combined risk profiles as disease progresses from steatosis to fibrosis, cirrhosis, and malignancy [16,31,34].

With respect to MetS, this systematic review found that MetS was infrequently examined as an exposure in relation to HCC risk in TX-based studies, with most investigations focusing on its individual cardiometabolic components. Garza et al. examined MetS in relation to cancer occurrence among Mexican Americans in south TX, reporting MetS was associated with a higher likelihood of cancer in relatives; however, outcomes were self-reported and not specific to HCC alone [35]. Similarly, Gill et al. reported a higher prevalence of MetS among individuals with NAFLD that was subsequently associated with subclinical atherosclerosis, but MetS itself was described descriptively rather than modeled as an exposure for liver disease progression or HCC outcomes [35]. Contextualizing this gap, a large non-TX based cohort study by Heiss et al. demonstrated MetS is highly prevalent in Hispanic/Latino/a adults, affecting approximately one third of the population, with prevalence exceeding 50% by midlife and rising further with age; Mexican origin participants exhibited MetS prevalence comparably to the overall cohort, and abdominal obesity was nearly universal amongst women with MetS. However, MetS was defined using standard U.S. waist circumference measurements (≥102 cm for men and ≥88 cm for women), thresholds that were developed largely in non-Hispanic White populations, and authors note the use of this threshold may inadequately capture central obesity risk in Hispanic populations and lead to misclassification [47]. This potential misclassification highlights an important methodological limitation that may contribute to heterogeneity across studies and future TX-based studies investigating Mexican American, Hispanic, and Latino/a populations can more rigorously define and model MetS exposure when evaluating HCC risk.

Evaluating earlier stages of liver disease alongside metabolic dysfunction was frequently present among individuals with NAFLD, steatosis, and fibrosis, including younger adults and first-degree relatives of Mexican Americans with HCC, suggesting that cardiometabolic risk factors occur well before advanced liver disease is clinically recognized [33,36]. However, the literature largely evaluates disease stages in isolation across separate cohorts, registries, and study designs rather than tracking longitudinal continuity from early metabolic dysfunction driving early liver disease clinical markers through subsequent cirrhosis and HCC development, restricting the ability to characterize timing and cumulative risk factor exposure effects [16,31,36]. Within studies measuring the isolated liver disease stages, related to early or advanced clinical markers, T2D and obesity as comorbidities and risk factors remained consistently associated with worsened liver disease prognosis, with only some of the literature incorporating additional risk factors such as alcohol or hepatitis exposures into their analysis [16,31,34]. For example, Jiao et al. evaluated diabetes and central obesity in relation to cirrhosis and advanced fibrosis while also examining HCV status, alcohol exposure, and PNPLA3 risk alleles [34] and Turner et al. evaluated the contribution of T2D and obesity to advanced liver disease at HCV diagnosis [16]. Despite these examples, limited research has examined the full set of metabolic, genetic, and environmental exposures together in a way that supports integrated risk. Similarly, while several studies identified T2D and obesity as a strong indicator of HCC risk [4,16,31,34,35,37] the underlying mechanism of disease progression specific to this population remains unclear.

Additionally, there is variability in measurement tools and outcome definitions used to characterize metabolic dysfunction and liver disease pathology across studies, including outcomes related to population-level incidence rates [6], imaging-based assessment of steatosis and fibrosis [36], and non-invasive fibrosis indices such as APRI [34] and FIB 4 [34]. The absence of standardized measurement approaches limits comparability across liver disease stages which reinforces the need for more consistent analytic frameworks that capture metabolic risk factors, co-occurring exposures, and liver disease outcomes across the disease pathology. These findings demonstrate consistent associations between metabolic dysfunction and liver disease burden, while emphasizing gaps in multirisk factor interaction analysis, longitudinal continuity across pathology stages, and standardized measurement that would strengthen interpretation of how metabolic exposures jointly contribute to HCC risk amongst south TX Mexican American, Hispanic, and Latino/a populations [4,6,16,31,33,34,35,36,37].

### 4.2. Environmental, Dietary, and Gene–Environment Contributions to HCC Risk

Environmental exposures, particularly alcohol consumption and aflatoxin contamination, were identified as modifiable risk factors for HCC among TX Hispanic/Latino populations; however substantial evidence gaps remain regarding exposure characterization, dietary patterns and gene–environment interactions. While alcohol consumption and aflatoxin biomarkers were documented across multiple studies [7,38,40,41,45], the evidence presented remains limited in its ability to define precise exposure characterization, including dose–response relationships, duration of exposure, and frequency of consumption, that could inform tailored prevention approaches [25,26]. Understanding these exposure characteristics is critical for designing effective public health interventions, as alcohol-related HCC risk operates through both direct hepatotoxic mechanisms and synergistic interactions with viral hepatitis, metabolic dysfunction, and genetic susceptibility [25,26].

Among the studies specific to alcohol consumption, this scoping review found that alcohol was infrequently examined with detailed exposure characterization in relation to HCC risk in TX-based studies, with most investigations providing only categorical classifications (e.g., “current heavy” vs. “not current heavy”) rather than quantified dose, duration, frequency, or drinking patterns. Among studies examining alcohol and HCC risk, only Gudenkauf et al. (2020) [35] provided population-level estimates of alcohol-attributable cancer burden (3.0% of cancers among Hispanics), while Thrift et al. (2024) [36] evaluated broad alcohol categories without detailed quantification of cumulative lifetime exposure or temporal drinking patterns. This represents a critical gap, as evidence from general populations demonstrates that both cumulative lifetime alcohol exposure and specific drinking patterns (binge drinking vs. chronic daily consumption) independently influence HCC risk through distinct pathophysiologic mechanisms, including direct hepatocyte injury, immune dysregulation, oxidative stress, and epigenetic modifications that persist even after alcohol cessation [25,26]. Understanding precise dose–response relationships is essential for developing risk-stratified HCC surveillance guidelines and targeted prevention interventions, as individuals with moderate-to-heavy cumulative alcohol exposure may benefit from intensified screening even in the absence of cirrhosis [11]. Furthermore, while Thrift et al. (2024) [36] identified synergistic interactions between heavy alcohol consumption and PNPLA3 genetic variants, no studies examined whether alcohol-metabolizing enzyme polymorphisms, such as ADH1B and ALDH2 variants that differ substantially in frequency across ethnic groups and alter acetaldehyde accumulation and hepatotoxicity, modify HCC risk among Hispanic/Latino populations [2]. A recent study by Tadokoro et al. (2025) demonstrated that ADH1B and ALDH2 variants significantly modify alcohol-related cirrhosis risk in Asian populations, yet similar genetic epidemiology studies remain absent for U.S. Hispanic communities, despite known differences in allele frequencies that could influence both individual susceptibility and population-level disease burden [48]. The absence of detailed alcohol exposure data and pharmacogenetic investigation limits the development of precision medicine approaches and risk stratification models that could identify high-risk Hispanic/Latino individuals who would benefit most from intensive surveillance, pharmacologic interventions, or behavioral alcohol reduction programs [11].

Similarly, while aflatoxin exposure was documented through biomarker detection (serum AFB_1_-lysine adducts, urinary AFM_1_) and molecular signatures (TP53R249S mutations) across multiple studies, few investigations directly examined dietary pathways, specific food sources, or consumption patterns that mediate aflatoxin exposure in TX Latino communities [7,39,40]. Pollock et al. (2016) [42] noted that Mexican Americans in south TX consumed corn tortillas significantly more frequently than the national average (56% vs. 20% consuming daily), suggesting a potential dietary exposure route; however, no studies systematically evaluated aflatoxin contamination levels in locally consumed foods, including masa, corn products, dried chilies, beans, or other staple foods prevalent in traditional Latino diets. This represents a substantial knowledge gap, as aflatoxin contamination patterns vary geographically based on agricultural practices, grain storage conditions, humidity and temperature during post-harvest storage, and regional food supply chain infrastructure, and direct measurement of dietary aflatoxin exposure through validated food frequency questionnaires coupled with targeted food contamination testing would better characterize population-level risk and identify intervention targets [27,41].

Evaluating dietary patterns beyond aflatoxin exposure revealed a near-complete absence of evidence, as no studies in this review directly examined habitual dietary intake, specific nutritional components, or dietary interventions in relation to HCC risk among TX Latino populations [7,27]. This gap is particularly concerning given that diet represents a modifiable risk factor with established protective effects against HCC in general populations, including consumption of coffee (reduces fibrosis progression and HCC incidence), vegetables and fruits (provide antioxidants and anti-inflammatory phytochemicals), whole grains (improve insulin sensitivity), and omega-3 fatty acids (reduce hepatic inflammation and steatosis) [17,24]. Notably, Ramirez et al. (2017) [45] reported that HCC cases were significantly less likely to be taking omega-3/fish oil supplements compared to matched controls, suggesting potential protective effects; however, habitual dietary intake of fish, nuts, and other omega-3 sources was not assessed, nor were other dietary components evaluated. Traditional Hispanic/Latino dietary patterns, which may include both protective elements (e.g., beans providing fiber and plant protein, fresh vegetables and fruits, fermented foods) and risk factors (e.g., sugar-sweetened beverages contributing to metabolic dysfunction, fried foods increasing oxidative stress), remain unexamined in relation to liver disease progression and HCC risk within this population [17]. The absence of dietary intervention studies is particularly striking given the high prevalence of metabolic dysfunction documented in Table 3, as dietary modification represents a cornerstone of MASLD management and has demonstrated efficacy in reducing hepatic steatosis, improving insulin sensitivity, and potentially reducing HCC risk in non-Latino populations [14].

Socioeconomic factors and acculturation, which influence both environmental carcinogen exposure and access to protective dietary patterns, remain critically understudied, as no studies evaluated how social determinants mediate differential exposure or modify HCC risk trajectories among Latino populations [11]. The near-complete absence of multicomponent dietary or environmental intervention trials limits the evidence base for comprehensive HCC prevention strategies within this high-risk population, despite the documented co-occurrence of multiple modifiable risk factors [14].

### 4.3. Intervention Evidence Across the HCC Care Continuum

Across the HCC care continuum, limited but informative intervention evidence demonstrates that targeted, context-specific strategies can improve screening, exposure reduction, and cardiometabolic risk management among predominantly Hispanic/Latino populations in TX. Two intervention studies demonstrated effective program interventions that increased screening numbers in predominantly Hispanic and Latino populations served by TX safety-net hospitals [42,43]. Taylor et al. implemented EMR-based identification of individuals born from 1945–1965 to screen for HCV exposure and chronic infection, coupled with patient navigation delivered by a *promotora* in outpatient settings to address barriers to care, communicate results to clinicians and coordinate follow-up [42]. Singal et al., similarly, reported improved screening outcomes through the integration of patient navigation alongside mailed outreach, facilitating linkage of eligible patients to specialized providers [43]. In both studies, hepatitis-related infection was identified as a common underlying liver disease etiology within these TX Hispanic cohorts, underscoring the continued relevance of hepatitis-focused screening and linkage-to-care strategies in efforts to reduce progression to HCC [42,43].

Contextualizing these findings, despite the emerging success of antiviral therapies, hepatitis-related infection remained the primary driver of HCC in Western countries at the times these studies were conducted [42,43]. This pattern is reflected in two TX-based intervention studies, where Singal et al. found the majority of cirrhosis patients undergoing screening presented with HCV-related cirrhosis across treatment arms. Taylor et al. reported HCV exposure in approximately 8% of their predominantly Mexican American cohort, substantially higher than national estimates for populations of similar race [42,43]. They further contextualized these results using population-based survey data, noting that HCV antibody prevalence varies by country of origin or ancestry, with higher rates reported among individuals of Puerto Rican descent than those of Mexican descent nationally [49]; however, their south TX Mexican American cohort demonstrated HCV prevalence comparable to that observed in Puerto Rican populations [42]. This distinction highlights the need to consider underlying demographic and other structural factors when interpreting why HCV antibody prevalence in TX cohorts may parallel levels reported in other racial and ethnic populations in different U.S. regions.

Furthermore, during the period these studies were published (2016–2017), metabolic factors were generally regarded as contributory rather than dominant etiologies of HCC [50]. By the latter half of the decade, however, evidence has indicated a shift in HCC etiology away from viral hepatitis toward metabolic-associated risk factors, particularly in southern U.S. states such as TX [51]. Other population-level analyses further contextualize this transition by examining HCC etiology across further Hispanic/Latino ethnic subgroups in other U.S. states. Pinheiro et al. reported overall declines in HCV-related HCC alongside increasing alcohol- and NAFLD-associated cases; however, HCV remained a major contributing etiology among specific subgroups, including Puerto Rican, African American, and U.S.-born Mexican men [52]. Collectively, these findings parallel earlier TX-based observations and suggest shared risk profiles across geographically distinct Hispanic/Latino populations, highlighting the need for continued attention to persistent and overlapping etiologies as HCC risk factors continue to shift over time.

Importantly, as HCC etiology has shifted over time toward metabolic-associated risk factors, evidence supporting upstream, non-viral intervention strategies remain limited. One community-based intervention study evaluated chronic disease management as a potential upstream prevention approach [37]. Lopez et al. demonstrated that a community-health-worker-integrated chronic care management program significantly improved blood pressure control among Hispanic/Latino adults with poorly controlled T2D and HTN in south TX, indicating the potential of CHW-based diabetes and cardiometabolic management as an upstream risk modification strategy to mitigate long-term liver disease and HCC risk, although liver outcomes were not directly assessed [37].

Evidence supporting targeted environmental exposure reduction is similarly limited but promising. Pollock et al. (2016) [42] demonstrated that low-dose calcium montmorillonite clay (ACCS100, 1.5 g/day) was effective in reducing aflatoxin bioavailability, as evidenced by significant reductions in serum AFB_1_-lysine adduct levels by month 3 (*p* = 0.0005) in a south TX Hispanic population, with no adverse safety signals observed in serum biochemistry or hematology. Although they did not mention the HCC outcomes, this intervention’s effectiveness in this high-exposure population suggests that dietary aflatoxin reduction strategies may represent a feasible, low-cost approach to mitigating HCC risk in vulnerable Latino communities chronically exposed to this hepatocarcinogen [41].

Pharmacologic prevention strategies remain largely unexplored. Only one article included in the present systematic review considered pharmacological management of diabetes in relation to HCC outcomes, reporting metformin use among Mexican Americans with T2D and HCC was associated with improved survival, suggesting a potential role for diabetes management in modifying disease course rather than primary intervention [38]. Metformin use was defined categorically, with no information on dose, timing, or duration of usage provided [38]. Additionally, no additional studies examined metformin or other glucose-lowering or incretin therapies as prevention considerations or strategies for HCC among Mexican American, Hispanic, or Latino/a populations in TX.

Across intervention-focused studies, structural barriers, including insurance status, fragmented care, and limited longitudinal follow-up, were frequently noted by respective authors [16]. Ramirez et al. (2017) [45] reported that HCC cases were significantly more likely to have Medicare or Medicaid insurance, lower income, and less education than controls, but the study did not explicitly test whether insurance status modified the association between environmental exposures (aflatoxin, alcohol) and HCC risk or whether adjusting for insurance attenuated observed risk estimates. In Turner et al., where insurance was used as a covariate adjustment, insurance status did not explain the elevated odds of liver advanced liver disease among Hispanics and Latinos with diabetes and obesity [16], suggesting other factors related to limitations in screening and diagnostic practices rather than access to coverage and care alone may be contributing to the disparities seen in this population.

### 4.4. Future Directions and Translational Implications

The evidence gaps and research priorities identified in this scoping review point toward several critical directions for advancing HCC prevention science in TX Hispanic/Latino communities. At the exposure characterization and epidemiologic level, prospective longitudinal studies with detailed quantitative assessment of alcohol consumption, dietary intake, and occupational/environmental exposures are urgently needed to track disease progression from early metabolic dysfunction and NAFLD/MASLD through cirrhosis and HCC development. Such studies should employ integrated multiomics approaches (genomics, transcriptomics, proteomics, metabolomics) to elucidate synergistic pathways among multiple simultaneous exposures and genetic predisposition, moving beyond isolated examination of single risk factors to characterize the complex etiology of HCC in this population. At the healthcare system and implementation level, evaluation of system-level interventions, including integrated primary care models combining metabolic disease management with HCV screening, telemedicine approaches to address rural healthcare access, and employment of culturally concordant providers and community health workers, should assess impact on HCC detection stage, treatment access, and ultimately HCC incidence and mortality outcomes among Hispanic/Latino populations [42,43]. Intervention research should address the heterogeneity of HCC etiology across Hispanic/Latino subgroups, recognizing that HCV-related HCC burden differs substantially by country of origin and that alcohol- and metabolic-associated HCC are rising in southern states including TX, requiring tailored prevention strategies for specific demographic subgroups [42,52]. Critically, these future directions should prioritize the development of multirisk stratification models tailored specifically to TX Hispanic/Latino populations to improve early detection and reduce the disproportionate burden of HCC. Given the identified synergy between metabolic dysfunction, genetic predisposition, diet, and environmental factors, a one-size-fits-all screening approach is insufficient. Transitioning toward personalized risk scores that integrate these interconnected variables will allow for more precise identification of high-risk individuals within this vulnerable population. Ultimately, translating these findings into community-engaged, culturally relevant screening protocols is essential for shifting the paradigm from late-stage diagnosis to effective prevention and early intervention.

### 4.5. Limitations and Strengths

This scoping review has some limitations that should be considered when interpreting its findings. The majority of studies included were cross-sectional or short-term prospective in design, with relatively few longitudinal investigations tracking liver disease progression, metabolic dysfunction, or HCC development over extended follow-up periods. This limits the ability to infer temporal relationships or causality, particularly for complex, multifactorial exposures such as diet, alcohol consumption, metabolic dysfunction, and gene–environment interactions that evolve with time.

Although genetic and molecular drivers of hepatocarcinogenesis were frequently examined, there remains a limited understanding of how genetic factors may interact with environmental and lifestyle exposures in Hispanic populations. Gene–environment interactions were rarely evaluated, and when assessed, analyses were often restricted to single genetic variants or broad exposure categories, limiting insight into synergistic or cumulative effects relevant to HCC risk. Additionally, current studies that include the TP53R249S gene have smaller tumor samples that could question the generalizability of the findings.

The populations represented across studies were predominantly Mexican American, reflective of the population in south TX, with minimal inclusion of other Hispanic/Latino subgroups. Given known heterogeneity in genetic ancestry, dietary patterns, alcohol metabolism, metabolic risk profiles, and socioeconomic context across Hispanic subgroups, these findings may not be generalizable to all Latino populations in the United States.

A notable limitation of the reviewed literature is the near-complete absence of studies examining dietary patterns, dietary interventions, traditional Latino foods, or acculturation-related dietary shifts in relation to HCC risk. No studies directly assessed habitual dietary intake using validated dietary assessment tools, nor did any evaluate how generational or acculturation-driven changes in diet may modify metabolic dysfunction or genetic susceptibility to HCC. This represents a critical gap, as diet is a modifiable exposure with established relevance to liver disease progression and metabolic health.

Additionally, potential misclassification of metabolic syndrome (MetS) and cardiometabolic risk may have occurred, as most studies relied on standard U.S waist circumference thresholds and diagnostic criteria developed in largely non-Hispanic White populations. These criteria may inadequately capture central adiposity and cardiometabolic risk among Hispanic populations, potentially underestimating the contribution of metabolic dysfunction to HCC risk.

Finally, much of the existing evidence on HCC etiology within Hispanic populations is derived from hospital-based cohorts, electronic medical records, or biobanks and databases. These data sources are subject to referral, insurance-related and healthcare-access-driven selection biases, which may underrepresent populations disproportionately affected by HCC. Consistent with prior reports, several studies noted challenges in recruitment, retention, and follow-up, even within safety-net hospital systems (Taylor 2016 [40], Singal 2017 [37]). While outreach-based screening interventions improved initial engagement and linkage to care, treatment uptake and longitudinal follow-up remained substantially lower, reflecting persistent structural barriers such as fragmented care, limited access to specialty services, and socioeconomic constraints. Collectively, these limitations highlight the need for population-based and community-engaged research approaches that extend beyond hospital-centered data sources and more accurately capture the combined effects of environmental, metabolic, genetic, and structural determinants of HCC risk in Hispanic communities.

Strengths

This scoping review demonstrates several methodological and substantive strengths that support robust synthesis of HCC risk factor evidence in TX Hispanic populations. The review employed rigorous systematic methods following PRISMA-ScR guidelines with comprehensive database searches across different platforms (PubMed, EbscoHost) yielding 20 high-quality studies. Standardized data extraction using consistent PICOS frameworks across all five summary tables ensured uniform capture of study characteristics, exposures, outcomes, and effect estimates. The inclusion of multiple study designs provides diverse evidence types and clean identification of evidence gaps to inform future research across metabolic, environmental, viral, genetic, and interventional studies. A key strength lies in the geographic and racial/ethnic specificity of the review: the concentration of studies within TX, particularly south Texas and the Rio Grande Valley, enabled detailed examination of region-specific HCC risk patterns and allowed comparison across multiple risk factor domains within the same well-characterized populations (primarily the Cameron County Hispanic Cohort). Finally, by synthesizing evidence across major risk factor categories, providing a holistic view of exposure characterization, dietary investigation, multirisk factor interactions, and intervention research, this review contributes to an actionable roadmap for future research, prevention strategies, and targeted interventions designed specifically for the TX Hispanic population.

## 5. Conclusions

Collectively, the findings of this systematic review indicate that HCC risk among Hispanic/Latino/a populations in TX may arise from the convergence of metabolic dysfunction, environmental exposures, viral infection, and structural determinants across the disease continuum. Although metabolic risk factors are consistently present across disease progression, evidence addressing their interaction with environmental, genetic, and healthcare system factors remains limited, constraining translation into prevention strategies. Future efforts should prioritize integrated and effective, culturally responsive prevention, screening, and intervention strategies in high-risk communities to reduce HCC burden in these communities.

## Figures and Tables

**Table 1:** Inclusion and exclusion criteria based on the PICOS framework.

	Inclusion Criteria	Exclusion Criteria
**Population (P)**	Hispanic/Latino adults (≥18 years) and with liver cancer.	Children and teenagers with cancer, non-Hispanic adults, other subgroups with no hx of liver cancer.
**Intervention (I)** **or Exposure (E)**	Aflatoxin Alcohol Diabetes Diet Genetics Hepatitis B/C Virus Metabolic Syndrome Obesity	No mention of aflatoxin. No mention of alcohol. No mention of diabetes. No mention of diet. No mention of Genetics. No mention of Hepatitis B/C Virus. No mention of Metabolic Syndrome. No mention of obesity.
**Control (C)**	N/A	N/A
**Outcome (O)**	Association with liver cancer, worse prognosis	No HCC or progression
**Study Design (S)**	Primary and secondary research; varied quantitative study designs, including experimental studies and observational studies with human participants.	Non-research reports (e.g., letters to the editor or editorials, commentaries, perspectives, protocols); qualitative studies.

**Table 2:** Overview of studies included in the scoping review.

Author, Year	Article Title	Risk Factor	Location	Study Design	Age [yo]	Mean Age [yo]	Population [N]	Sexes		Race/Ethnicity	City/County	Intervention	Intervention Type/Dosing
Das et al., 2024 [5]	Integrative Multi-omics Characterization of HCC in Hispanic patients	Genetics	South TX	Observational	Adults ≥ 40	NR	109 ^a^	F M	42 ^a^ 67 ^a^	100% Hispanic	San Antonio	No	NA
El-Serag et al., 2021 [6]	TX has the Highest HCC Rates in the US	DM MetS Obesity	TX and US	Observational (2001–2015) *TCR* and *NPCR-SEER*	Adults ≥ 25	NR	2481	F M	612 1869	Mex. American: 39.5% Hispanic (n = 981)	32 counties w/in 100 miles of the US/Mex. border	No	NA
Garza et al., 2016 [33]	Liver and Other GI Cancers are Frequent in Mex. Americans	DM MetS Obesity	RGV	Cross-Section Observational (2004–2014) *CCHC*	Adults ≥ 18	45.2	2554	F M	1692 862	Mex. American: 100% Hispanic	Brownsville Cameron County	No	NA
Gill et al., 2017 [38]	Frequency of NAFLD and Subclinical Atherosclerosis Among Young Mex. Americans	DM MetS Obesity	RGV	Cross-Section Observational *CCHC*	Adults ≥ 18	50.4	407	F M	237 ^a^ (58.3%) 170 ^a^ (41.7%)	Mex. American: 100% Hispanic	Brownsville Cameron County	No	NA
Gudenkauf et al., 2020 [35]	Preventable causes of cancer in TX by race/ethnicity: Alcohol consumption	Alcohol	TX	Observational *TCR-SEER*	Adults ≥ 25	NR	103,408 ^b^	F M	NR NR	(NR) Hispanic	Statewide	No	NA
Hatia et al., 2025 [31]	Risk and Prognosis of HCC in Mex. Americans with T2D	Alcohol HBV/HCV DM	South TX	Case–Control Observational (2000–2020) *Mano y Mano*	Adults ≥ 18	61.4 ^a^	741	F M	226 ^a^ 515 ^a^	Mex. American: 100% Hispanic	Houston Harris County	No	NA
Jiao et al., 2016 [34]	Cirrhosis and Advanced Fibrosis in Hispanics in TX: the Dominant Contribution of Central Obesity	Alcohol DM Genetics HCV Obesity	RGV	Observational (2004–2015) *CCHC*	Adults ≥ 25	46 ^a^	2466	F M	1393 ^a^ (56.5%) 1073 ^a^ (43.5%)	Mex. American: 100% Hispanic	Brownsville Harlingen Cameron County	No	NA
Jiao et al., 2018 [7]	Prevalence of Aflatoxin-associated TP53R249S Mutation in HCC in Hispanics in South TX	Aflatoxin Genetics	South TX	Cross-Sectional, Mutation Detection (2002–2010) ^c^ *CCHC*	Adults ≥ 30–88 ^d^	63 ^a^	314 ^a^	F M	58 ^a^ (18.4%) 256 ^a^ (81.6%)	Mex. American: 100% Hispanic	Houston (Multiple Counties)	No	NA
Jiao et al., 2021 [41]	Somatic Mutations in Circulating Cell-Free DNA and Risk for HCC in Hispanics	Genetics	South TX	Observational *CCHC*	Adults ≥ 50.4	56.4 ^a^	119 ^a^	F M	62 ^a^ 57 ^a^	100% Hispanic	NR	No	NA
Lee et al., 2021 [4]	State-Level HCC Incidence and Association with Obesity and PA in the US	Obesity PA	US	Observational (2001–2017) BRFSS (2011–2017)	NR	NR	299,116	F M	NR NR	Mex. American: 15.6% Hispanic	State Level	No	NA
Lopez et al., 2024 [39]	An Expanded Chronic Care Management Approach to Multiple Chronic Conditions in Hispanics Using Community Health Workers as Community Extenders in the RGV of TX	T2D	RGV	Multivariate Longitudinal Intervention *Salud y Vida Program* (2013–2020)	Adults ≥ 18	52.89	3806	F M	2702 ^a^ (71%) 1104 ^a^ (29%)	Mex. American: 100% Hispanic	Brownsville Cameron County	Yes	CHW + DSME
Ma et al., 2022 [46]	Autoantibody Against Tumor-Associated Antigens as Diagnostic Biomarkers in Hispanic Patients with HCC	Genetics	TX	Translational (Case–Control and In Vitro Approach)	NR	NR	227	F M	NR NR	48.5% Hispanic	El Paso	No	NA
Pollock et al., 2016 [42]	Intervention Trial with Calcium Montmorillonite Clay in a South TX Population Exposed to Aflatoxin	Aflatoxin	South TX	Double-Blind Placebo-RCT	Adults 18–77	NR	234	F M	180 54	Predominantly Latino/Hispanic	Bexar and Medina Counties	Yes	ACCS100: Placebo, LD (1.5 g/d), HD (3 g/d) for 3 m
Ramirez et al., 2017 [45]	Lifestyle and Clinical Correlates of HCC in South TX: A Matched Case-Control Study	Aflatoxin Alcohol Lifestyle	South TX	Matched Case–Control	Adults ≥ 18	NR	155 ^a^	F M	NR NR	67% Latino	Bexar County and 7 Surrounding Counties	No	NA
Sharpton et al., 2023 [44]	Prevalence and Factors Associated with Liver Fibrosis Among First-Degree Relatives of Mex. Americans with HCC	T2D Obesity	RGV	Cross-Sectional Prospective *HLCC-CCHC*	Adults ≥ 18	50.3	182	F M	106 ^a^ (58%) 76 ^a^ (42%)	Mex. American: 100% Hispanic	Brownsville Cameron County	No	NA
Singal et al., 2017 [37]	Mailed Outreach Program Increases Ultrasound Screening of Patients with Cirrhosis for HCC	HBV	TX	Prospective/Intervention (Mailed Outreach)	Adults ≥ 21.2	55.3	1800	F M	731 ^a^ (40.6%) 1069 ^a^ (59.4%)	37.8% Hispanic	Dallas County	No	NA
Taylor et al., 2016 [40]	Hospital-Based HCV Screening of Baby Boomers in a Majority Hispanic South TX Cohort: Successes and Barriers to Implementation	HCV	TX	Intervention (Baby Boomer Screening Program)	Adults ≥ 55.5	58	2327	F M	1021 (44%) 1306 (56%)	59% Hispanic	San Antonio	No	NA
Thrift et al., 2023 [43]	Risk Stratification for HCC Among Patients with Cirrhosis Using a Hepatic Fat Polygenic Risk Score	Genetics	TX	Observational THCCC, HVASC	Adults ≥ 55–65	59.8	1644	F M	517 1127	27.2% Hispanic	Dallas Fort Worth Houston McAllen San Antonio	No	NA
Thrift et al., 2024 [36]	PNPLA3, Obesity, and Heavy Alcohol Use in Cirrhosis Patients May Exert a Synergistic Increase in HCC Risk	Alcohol Genetic Obesity	TX	Prospective Cohort THCCC	Adults ≥ 49.5	59.6	1911	F M	682 ^a^ (35.7%) 1229 ^a^ (64.3%)	28.7% Hispanic	Dallas Galveston Houston San Antonio	No	NA
Turner et al., 2019 [16]	Significant Increase in Risk of Fibrosis or Cirrhosis at Time of HCV Diagnosis for Hispanics with DM and Obesity Compared with Other Ethnic Groups	Alcohol T2D Obesity	TX	Cross-Section Observational (2015–2017)	Adults born from 1945–1965	58	748	F M	255 (34.1%) 493 (65.9%)	Mex. American: 21.8% Hispanic	North and South (6 Health Systems)	No	NA

^a^ Calculated for this scoping review from data provided in the reference. ^b^ Study reported total TX cancer cases (excluding BCC and SCC of the skin) diagnosed in 2015 in adults aged ≥ 25 years; study did not report a demographics table. ^c^ Study reported only for HCC tumor: other cohort years not stated. ^d^ Study period reported only for plasma cfDNA samples (2002–2010); other cohort years not stated. Abbreviations: ACCS100, Calcium Montmorillonite Clay; CCHC, Cameron County Hispanic Cohort; CHW, Community Health Worker; DM, Diabetes Mellitus; DSME, Diabetes Self-Management Education; F, Female; g/d, grams/day; GI, Gastrointestinal; HCC, Hepatocellular Carcinoma; HBV, Hepatitis B Virus; HCV, Hepatitis C Virus; HD, High-Dose; HLCC, Hispanic Liver Cancer Cohort; HVASC, Houston Veterans Administration Cirrhosis Surveillance Cohort; LD, Low-Dose; M, Male; MetS, Metabolic Syndrome; Mex., Mexico; NA, Not Applicable; m, Months; NAFLD, Non-Alcoholic Fatty Liver Disease; NPCR-SEER, U.S. National Program of Cancer Registries and Surveillance, Epidemiology, and End Results Program; NR, Not Reported; PA, Physical Activity; RCT, Randomized Controlled Trial; RGV, Rio Grande Valley; TCR, Texas Cancer Registry; THCCC, Texas Hepatocellular Carcinoma Consortium Cohort; T2D, Type 2 Diabetes; TX, Texas; U.S., United States; yo, Years Old.

**Table 3:** Summary of metabolic-dysfunction-related risk factors found across studies: diabetes, metabolic syndrome, and obesity.

Author, Year	Article Title	Location	Registry	Years Analyzed	Objective/Exposure	Measurable Outcomes	Key Findings
El-Serag et al., 2021 [6]	TX has the Highest HCC Rates in the US	TX US	TCR NPCR + SEER	2001–2015	To determine whether HCC incidence in TX differs from U.S. trends and varies by sex, race, ethnicity, age, or region of TX.	HCC incidence rate (age adjusted, per 100,000 population).	TX had the highest HCC incidence rate in the U.S. in 2015 (13.2 per 100,000); 45% higher than the national average. HCC incidence increased over time from 2001 to 2015 in both TX and the U.S. at ~4% per year. Rates were consistently higher in TX than nationally across sex, race ethnicity, and age groups. M had ~3× higher incidence than F, but both sexes showed increasing trends. Hispanics in TX had the highest HCC incidence, higher than Hispanics in all other U.S. states. Middle aged and older adults (55–74 years) had the highest incidence rates, with the largest increases over time. South TX and U.S.–Mexico border regions had higher HCC incidence than the rest of TX.
Garza et al., 2016 [33]	Liver and Other GI Cancers are Frequent in Mex. Americans	RGV	CCHC	Recruitment began 2004	To determine the frequency of GI cancers in Mex. Americans and assess associations with metabolic risk factors including DM, obesity, and MetS.	Self-reported cancer occurrence in participants and first- and second-degree relatives.	Among 9249 individuals, 1184 cancer cases were reported across participants and their first- and second-degree relatives. DM was the strongest risk factor for cancer among cohort participants under age 70 (OR 3.57, 95% CI: 1.32–9.62). MetS was associated with higher likelihood of cancer in relatives, including increased odds when one or more parents or siblings had cancer. GI cancers ranked unusually high among Mex. Americans, particularly liver and stomach cancers, in both mothers and fathers. The ranking of gastrointestinal cancers in this cohort resembled patterns seen in Mexico more than those seen in other U.S. populations. Local age adjusted cancer registry data confirmed higher incidence of liver and stomach cancers in CCHC compared with non-Hispanic populations.
Gill et al., 2017 [38]	Frequency of NAFLD and Subclinical Atherosclerosis Among Young Mex. Americans	RGV	CCHC	NR	To determine the prevalence of NAFLD and its association with subclinical atherosclerosis in Mex. Americans.	NAFLD prevalence by liver ultrasound; subclinical atherosclerosis measured by carotid intima media thickness (cIMT) and carotid plaque.	NAFLD was highly prevalent in Mex. American cohort (~49%). Participants with NAFLD had higher BMI, central obesity, fasting glucose, dyslipidemia, and were more likely to have MetS. Nearly one third of participants with NAFLD had evidence of subclinical atherosclerosis. After adjustment, NAFLD was independently associated with increased cIMT in younger participants <45 years, but not in older adults. Participants with both abnormal liver and carotid ultrasound findings tended to be obese, diabetic, and have MetS.
Hatia et al., 2025 [31]	Risk and Prognosis of HCC in Mex. Americans with T2D	South TX	MD Anderson study population; Mano a Mano Mex. American Cohort (controls)	January 2000–December 2020	To determine the risk and prognosis of HCC in Mex. Americans with T2D, including effects of DM duration, treatment, and interactions with alcohol use and viral hepatitis.	HCC risk (adjusted odds ratios); overall survival (hazard ratios).	T2D independently associated with increased HCC risk (OR 2.74, *p* < 0.01). Longer duration of DM showed a dose response, with ≥20 years associated with markedly higher HCC risk (OR 4.60). T2D interacted synergistically with viral hepatitis infection and heavy alcohol consumption to further increase HCC risk. Metformin use was associated with improved survival among HCC patients with T2D (HR 0.72, *p* = 0.01).
Jiao et al., 2016 [34]	Cirrhosis and Advanced Fibrosis in Hispanics in TX: the Dominant Contribution of Central Obesity	RGV	CCHC	2004–2015	To determine prevalence and associated risk factors for cirrhosis in Hispanic populations of south TX.	Clinical and demographic variables, AST to platelet ratio index (APRI) as predictor for cirrhosis (APRI ≥ 2; APRI ≥ 1).	Prevalence of cirrhosis (APRI ≥ 2) was 0.94%, nearly 4× higher than national estimates; prevalence of cirrhosis/advanced fibrosis (APRI ≥ 1) was 3.54%. Highest prevalence was observed in M, particularly ages 25–34 years. Independent risk factors for cirrhosis and/or advanced fibrosis include hepatitis C, DM, and central obesity. Central obesity accounted for the largest population attributable fraction (52.5% of cirrhosis; 65.3% of cirrhosis/advanced fibrosis). Excess alcohol consumption was independently associated with cirrhosis and contributed to earlier disease onset in males when combined with central obesity. PNPLA3 risk alleles were associated with higher APRI scores and increased odds of cirrhosis/advanced fibrosis, particularly in participants >50 years old.
Lee et al., 2021 [4]	State-Level HCC Incidence and Association with Obesity and PA in the US	US	NR	NR	Characterize state-level racial/ethnic disparity in HCC incidence, state-level temporal changes in HCC incidence, and ecological correlation between HCC incidence and obesity/physical activity levels in the U.S.	Sex, age, race/ethnicity (non-Hispanic White, Black, American Indian/Alaska Native (AI/AN), Asian/Pacific Islander (API), and Hispanic), and state. Obesity was defined as individuals with body mass index ≥30 kg/m^2^. PA was defined as individuals who achieve ≥150 min per week of moderate-intensity aerobic physical activity or ≥75 min per week of vigorous-intensity aerobic activity.	HCC incidence trends had a moderate correlation with state-level obesity and a moderate-inverse correlation with state-level physical activity. Incidence rates were highest in APIs (10.8/100,000 PY) followed by Hispanics (9.6/100,000 PY), AIs/ANs (8.5/100,000 PY), and Blacks (7.3/100,000 PY) and lowest in Whites (4.0/100,000 PY). State-level incidence rate ratio (IRR) between Hispanics and Whites is 2.6 in TX. The IRRs for Hispanics were highest in Minnesota (IRR 3.8) and lowest in Alabama (IRR 0.9). Variation in incidence rates between states continued to decrease through 2017, with an IRR of only 2.6 between states with the highest (TX at 8.8 per 100,000 PY) and lowest (New Hampshire at 3.4 per 100,000 PY) incidence.
Lopez et al., 2024 [39]	An Expanded Chronic Care Management Approach to Multiple Chronic Conditions in Hispanics Using Community Health Workers as Community Extenders in the RGV of TX	RGV	Salud y Vida Cohort	2013–2020	To determine the effect of a CHW-integrated expanded chronic care management intervention on BP outcomes among Hispanics with poorly controlled T2D and HTN.	Changes in systolic and diastolic blood pressure over time (mmHg).	Among 3806 Hispanic adults with poorly controlled T2D and hypertension, mean SBP and DBP decreased significantly from baseline to 3 months (SBP − 6.49 mmHg; DBP − 3.97 mmHg; both *p* < 0.001) and were sustained up to 24 months. Participants with higher program engagement had greater reductions in SBP at 3 months (−1.8 mmHg) and 15 months (−2.3 mmHg) compared with lower engagement. Both higher and lower engagement groups showed significant early BP improvement, but greater and more sustained SBP reduction was observed in the higher engagement group. Hispanics in the south TX region with co-occurring T2D and HTN experience fragmented care and require support navigating healthcare systems.
Sharpton et al., 2023 [44]	Prevalence and Factors Associated with Liver Fibrosis Among First-Degree Relatives of Mex. Americans with HCC	RGV	HLCC; ancillary to CCHC	NR	To determine the prevalence of significant hepatic fibrosis and steatosis in first-degree relatives of Mex. Americans with HCC and identify associated clinical factors.	Prevalence of significant hepatic fibrosis (LSM ≥ 7.0 kPa by VCTE); definite hepatic steatosis (CAP ≥ 288 dB/m); suspected cirrhosis.	Among 112 first-degree relatives, 17% had significant hepatic fibrosis and 42% had definite hepatic steatosis. Prevalence of fibrosis increased to 20% among first-degree relatives ≥ 40 years; 5% met criteria for suspected cirrhosis. T2D (OR 3.2) and AST ≥ 30 IU/L (OR 4.0) were independent predictors of hepatic fibrosis. Obesity, elevated ALT, and higher TG were strongly associated with hepatic steatosis. Findings suggest a high burden of clinically significant liver disease in first-degree relatives of Mex. Americans with HCC, supporting consideration of targeted screening.
Turner et al., 2019 [16]	Significant Increase in Risk of Fibrosis or Cirrhosis at Time of HCV Diagnosis for Hispanics with DM and Obesity Compared with Other Ethnic Groups	TX	EMR data from 6 healthcare systems and FQHCs	2015–2017	To determine whether metabolic risk factors (DM and obesity) contribute to racial ethnic disparities in advanced liver disease at time of HCV diagnosis and assess interactions with heavy alcohol use.	Advanced liver disease defined by FIB 4 > 3.25 at time of HCV diagnosis. ^a^	Advanced liver disease was present in 22.9% of patients at HCV diagnosis. Hispanics had higher odds of advanced liver disease than NHBs (OR 2.60) and NHWs (OR 1.94). Among patients with obesity and DM, Hispanics had markedly higher odds of advanced liver disease compared with NHBs (OR 7.89) and NHWs (OR 12.49). Heavy alcohol use and older age were independently associated with advanced liver disease. Findings indicate synergistic effects of Hispanic ethnicity, T2D, and obesity on advanced liver disease risk at HCV diagnosis.

^a^ Models were adjusted for age, sex, insurance status, and heavy alcohol use. Abbreviations: AI/AN, American Indian or Alaska Native; ALT, Alanine aminotransferase; APRI, Aspartate to platelet ratio index; API, Asian or Pacific Islander; AST, Aspartate aminotransferase; BMI, Body mass index; BP, Blood pressure; CAP, Controlled attenuation parameter; CCHC, Cameron County Hispanic Cohort; CHW, Community health worker; cIMT, Carotid intima media thickness; DM, Diabetes mellitus; EMR, Electronic medical record; FIB 4, Fibrosis index; FQHC, Federally qualified health center; GI, Gastrointestinal; HCC, Hepatocellular carcinoma; HCV, Hepatitis C virus; HLCC, Hispanic Liver Cancer Cohort; HR, Hazard ratio; HTN, Hypertension; IRR, Incidence rate ratio; LSM, Liver stiffness measurement; M, Male; MetS, Metabolic syndrome; NAFLD, Non-alcoholic fatty liver disease; NHB, Non-Hispanic Black; NHW, Non-Hispanic White; NPCR, National Program of Cancer Registries; NR, Not reported; PA, Physical activity; PNPLA3, Patatin-like phospholipase domain-containing protein 3; PY, Person-years; RGV, Rio Grande Valley; SBP, Systolic blood pressure; SEER, Surveillance, Epidemiology, and End Results Program; T2D, Type 2 diabetes; TCR, Texas Cancer Registry; TG, Triglycerides; TX, Texas; U.S., United States; VCTE, Vibration-controlled transient elastography.

**Table 4:** Summary of environmental-related exposure: Aflatoxin and alcohol.

Author, Year	Article Title	Location	Data Source	Years Analyzed	Objectives/Exposure	Measurable Outcomes	Key Findings
Gudenkauf et al., 2020 [35]	Preventable Causes of Cancer in TX by Race/Ethnicity: Alcohol Consumption	TX	TCR	2015	To estimate the percentage and number of cancer cases diagnosed in TX in 2015 that are attributable to alcohol consumption. To examine differences in estimates across major population racial/ethnic subgroups.	Weighted prevalence estimates of alcohol consumption; PAFs; RR calculated for alcohol consumption according to WCRF/AICR standards.	Alcohol consumption caused 2.9% of all cancers in TX [2974 cases] in 2015. Hispanic populations showed 3.0% attributable cases compared to 2.7% in non-Hispanic Whites and 2.2% in non-Hispanic Blacks. Men had higher attributable fractions [3.6%] than women [2.2%]. Alcohol consumption reported RR = 1.04 of developing liver cancer.
Jiao et al., 2018 [7]	Prevalence of Aflatoxin-associated TP53R249S Mutation in HCC in Hispanics in South TX	South TX (Cameron, Webb, Harris counties, Galveston)	CCHC	2002–2010	To examine the effect of aflatoxin exposure on development of HCC-related TP53R249S mutation in Hispanic populations and assess associations with other baseline risk factors including HCV.	Primary: TP53R249S mutation prevalence in HCC tumors and plasma cell-free DNA analyzed using droplet digital PCR and restriction fragment length polymorphism. Secondary: Association with survival outcomes and age at diagnosis.	TP53R249S mutation detected in 7.3% [3/41] of Hispanic HCC tumors and 5.7% of plasma cfDNA samples from Hispanic HCC patients. Patients with this mutation were significantly younger and had shorter overall survival [*p* < 0.05]. The mutation was detected only in Hispanic and Asian patients, never in non-Hispanic populations. Mutation associated with earlier onset and worse prognosis.
Pollock et al., 2016 [42]	Intervention Trial with Calcium Montmorillonite Clay in a South TX Population Exposed to Aflatoxin	Bexar and Medina Counties, TX	Primary data collection	2016 (3 m intervention)	To evaluate the effects of ACCS100 on reducing serum AFB_1_-lysine adduct levels and assess safety parameters in predominantly Hispanic, aflatoxin-exposed populations.	Primary: Serum AFB_1_-lysine adduct levels at baseline, 1, 3, and 4 m of intervention. Secondary: Safety parameters including serum biochemistry and hematology. Detection and quantification of serum AFB_1_-lysine adducts using laboratory analysis.	Low-dose ACCS100 [1.5 g/day] showed significant reduction in AFB_1_-lysine adduct levels by m 3 [*p* = 0.0005]. Among 234 participants [100% Hispanic; 180 females, 54 males; age range 18–77 years], Mexican Americans in the study region consumed corn tortillas significantly more frequently than the national average [56% vs. 20% consuming daily]. Use of ACCS100 demonstrated as viable strategy to reduce dietary AFB_1_ bioavailability during aflatoxin outbreaks and in chronically exposed populations.
Ramirez et al., 2017 [45]	Lifestyle and Clinical Correlates of HCC in South TX: A Matched Case-Control Study	South TX	Primary data collection	2000–2020	To determine relative etiologic contributions of lifestyle-related and clinical risk factors for HCC in south TX, including aflatoxin exposure, alcohol/tobacco use, healthcare access, and viral infections.	Primary: Comparison between HCC cases and matched controls among Latino (67%) participants. Clinical and lifestyle factors: health insurance status, income, education level, lifetime alcohol and tobacco use, past medical history (hypercholesterolemia, HCV, cirrhosis, blood transfusion), medication use (aspirin, statins, omega-3/fish oil), detection of HCV antibodies, and presence of aflatoxin biomarkers in blood and urine.	Cases showed higher rates of lifetime alcohol and tobacco use. HCC cases were significantly more likely to have Medicare or Medicaid, lower income, and less education than controls. Medical History Findings: Cases were less likely to have hypercholesterolemia [OR 0.11, 95% CI: 0.02–0.51]. Markedly more likely to report hepatitis C infection [OR: 183.74, 95% CI: 27.37–∞], cirrhosis [OR: 2.17, 95% CI: 33.3–∞], and history of blood transfusions [OR: 4.35, 95% CI: 1.60–11.84]. Medication and Supplement Use Findings: Cases were less likely to be taking aspirin [OR: 0.31, 95% CI: 0.11–0.85], statins [OR: 0.03, 95% CI: 0–0.20], or omega-3/fish oil supplements [OR: 0.10, 95% CI: 0.01–0.78]. No significant difference in reported consumption of corn products. Laboratory Findings: Cases were far more likely than controls to have HCV antibodies [OR 174.3, 95% CI: 26.2–∞]. Cases had higher odds of detectable aflatoxin biomarkers in blood [OR 6.09, 95% CI: 1.10–33.71] and urine [OR 3.42, 95% CI: 1.07–10.91].
Thrift et al., 2024 [36]	PNPLA3, Obesity, and Heavy Alcohol Use in Cirrhosis Patients May Exert a Synergistic Increase in HCC Risk	TX	THCCC + HVASC	2022	To examine whether germline susceptibility variants (PNPLA3 I148M) independently predispose to HCC and act synergistically with metabolic and behavioral risk factors (obesity, heavy alcohol use) in cirrhosis patients.	Primary: HCC development using Cox regression with competing risks. Classification by: alcohol consumption status (current heavy vs. not), BMI (≥30 vs. >30), and PNPLA3 I148M variant status (carrier of at least one G risk allele vs. non-carrier). Stratified analysis by genetic variant status, obesity, and alcohol consumption patterns.	PNPLA3 variant demonstrated synergistic effects: Carriers with heavy alcohol consumption had 2.65-fold higher HCC risk [HR 2.65, 95% CI: 1.20–5.86] compared to non-carriers without heavy drinking. Carriers with obesity had 2.40-fold higher risk (HR 2.40, 95% CI: 1.33–4.31, *p* < 0.05). Among 1911 cirrhosis patients (n = 1229 males, n = 682 females; 28.7% Hispanic; mean age 59.6 yo), synergistic effects were particularly pronounced in patients with concurrent viral hepatitis. PNPLA3 variant may help refine HCC risk stratification for patients with cirrhosis requiring specific preventive measures.

Abbreviations: AFB_1_, aflatoxin B_1_; AFB_1_-lysine, aflatoxin B_1_-lysine adduct; BMI, body mass index; CCHC, Cameron County Hispanic Cohort; cfDNA, cell-free DNA; CI, confidence interval; GI, gastrointestinal; HCC, hepatocellular carcinoma; HCV, hepatitis C virus; HR, hazard ratio; HVASC, Houston Veterans Administration Cirrhosis Surveillance Cohort; m, month; OR, odds ratio; PAF, population attributable fraction; PNPLA3, patatin-like phospholipase domain-containing protein 3; RGV, Rio Grande Valley; RR, relative risk; TCR, Texas Cancer Registry; TX, Texas; THCCC, Texas Hepatocellular Carcinoma Consortium Cohort; TP53R249S, aflatoxin-associated *TP53* codon 249 serine mutation; yo, years old.

**Table 5:** Summary of infection/virus-related exposure: Hepatitis B and Hepatitis C.

Author, Year	Article Title	Location	Registry	Years Analyzed	Objective/Exposure	Measurable Outcomes	Key Findings
Taylor et al., 2016 [40]	Hospital-Based Hepatitis C Screening of Baby Boomers in a Majority Hispanic South TX Cohort: Successes and Barriers to Implementation	San Antonio, TX	HCHS/SOL NHANES	2013–2014	To determine outcomes of implementing hepatitis C screening methods in Baby Boomer (1945–1965) patients in a south TX safety-net hospital (University Hospital, SA, TX).	Anti-HCV-positive status confirming proof of infection exposure. Current active HCV infection.	Eight-percent anti-HCV prevalence found in Hispanic people of this cohort, nearly four times the prevalence seen in Hispanics of Mexican descent reported in NHANES or HCHS/SOL.
Singal et al., 2017 [37]	Mailed Outreach Program Increases Ultrasound Screening of Patients with Cirrhosis for HCC	Dallas, TX	AASLD	December 2014–March 2016	To determine effectiveness of outreach strategies and patient support in increasing HCC screening participation in cirrhosis cohort within a large safety-net system (PHHS, Dallas, TX).	Increased one-time HCC screening participation. Decreased time-to-response to outreach invitations.	Hispanics were 1.56× more likely vs. NHW to participate in screening services (OR 1.56, 95% CI: 1.20–2.02). M sex was less likely to participate in screening, despite being target demographic for HCC (OR 0.80, 95% CI: 0.65–0.99). Increased age correlated with modest increase in participation in screening (OR 1.52, 95% CI: 1.20–1.93). Primary care contact (AOR 1.05, 95% CI: 1.03–1.08) and GE care (AOR 0.74, 95% CI: 1.35–2.21) were associated with increased screening rates.

Abbreviations: AASLD, American Association for the Study of Liver Diseases; AOR, Adjusted Odds Ratio; GE, Gastroenterologist; HCC, Hepatocellular Carcinoma; HCHS, Hispanic Community Health Study; HCV, Hepatitis C Virus; M, Male; NHANES, National Health and Nutrition Examination Survey; NHW, Non-Hispanic White; OR, Odds Ratio; SOL, Study of Latinos; PHHS, Parkland Health Hospital System.

**Table 6:** Summary of genetic risk and genetic predisposition exposure: Genetics.

Author, Year	Article Title	Location	Database/Registry	Years Analyzed	Objectives/Exposure	Measurable Outcomes	Key Findings
Das et al., 2024 [5]	Integrative Multi-Omics Characterization of HCC in Hispanic Patients	South TX	100 Genome Project CSMC ICGC PCWGC TCGA-LIHC	NR	To determine molecular alterations specific to HCC among Hispanic populations using a multiomics approach.	Whole-exome sequencing, TERT promoter sequencing, RNA sequencing, mass spectrometry analysis of proteomic data, metabolomic analysis, serum lipidomic analysis.	Higher rates of Wnt gene mutations in Hispanic cohort: *AXIN2* mutation frequency was significantly higher in south TX Hispanic HCC than NHW (11.1% vs. 0.6%; *p* = 0.00912). South TX Hispanic cohort had lower *TP53* mutation frequency and a higher *CTNNB1* mutation frequency than African American patients from TCGA-LIHC (*p* = 0.00032). Significantly higher rate of *TERT* promoter mutation (primarily C228T) in the south TX Hispanic HCC cohort than in TCGA-LIHC White (77.8% vs. 47.8%; *p* = 0.00535) and Asian (77.8% vs. 31.5%; *p* = 0.00012) patients.
Jiao et al., 2021 [41]	Somatic Mutations in Circulating Cell-Free DNA and Risk for HCC in Hispanics	South TX	MDA T200.1	NR	To identify somatic mutations in cfDNA of Hispanics with HCC vs. Hispanics with advanced liver fibrosis but no HCC.	APRI scores to determine liver fibrosis/cirrhosis, family and medical history for HCC incidence, T2D and alcohol consumption, blood samples for targeted gene sequencing.	*TP53* identified as most commonly mutated gene within this cohort (27%) followed by *NFE2L2* and *CTNNB1* (14%), *KMT2D*, *KMT2C*, *AXIN1*, *AR*, and *BIVM-ERCC5* (9%). Somatic mutations in these genes of interest tested for recruited study participants; *KMT2D* correlated (17.6%) with advanced liver fibrosis/cirrhosis.
Ma et al., 2022 [46]	Autoantibody Against Tumor-Associated Antigens as Diagnostic Biomarkers in Hispanic Patients with HCC	South TX	CARL-UTEP	NR	To investigate novel TAA autoantibodies as diagnostic biomarkers for Hispanic HCC patients.	TAA targets were identified by SERPA and from differentially expressed HCC driver genes via bioinformatics. ELISA used for validation.	p16, *SETDB1*, RNA helicase A, *BRG1*, GNAS, Merlin, *DNMT3A*, *NRAS*, GMPS, and *ERK2* identified as potential TAAs related to HCC driver genes in Hispanic HCC cohort. *DNMT3A* (45.8%), p16 (41.7%), HSP60 (37.5%), and *HSPA5* (33.3%) identified as significant TAAs in Hispanic cohort compared to NHS; suggested as potential diagnostic biomarkers for Hispanic HCC patients.
Thrift et al., 2023 [43]	Risk Stratification for HCC Among Patients with Cirrhosis Using a Hepatic Fat Polygenic Risk Score	South TX	AASLD HVASC THCCC	2016–2021	To evaluate the performance of a PRS, including variants in *PNPLA3*, *MBOAT7*, *TM6SF2*, and *GCKR*, for predicting risk of developing HCC in two contemporary U.S.-based multiethnic cohorts of patients with cirrhosis.	Development of HCC incidence after enrollment of cirrhotic patients, and genotyping of patients from multicenter cohorts using germline DNA. Documented anthropometric data along with sociodemographic and clinical risk factors (DM, DLD, HTN).	Frequency of G allele mutations in PNPLA3 was highest among Hispanics (65%).

Abbreviations: AASLD, American Association for the Study of Liver Diseases; APRI, AST to platelet ratio index; AR, Androgen receptor gene; *AXIN1*, Axis inhibition protein 1 gene; *AXIN2*, Axis inhibition protein 2 gene; *BIVM-ERCC5*, Readthrough transcript fusion gene; *BRG1*, Brahma-related gene 1; CARL-UTEP, Cancer Autoimmunity Research Laboratory—University of Texas El Paso; cfDNA, Plasma cell-free DNA; CSMC, Catalogue of Somatic Mutations in Cancer; *CTNNB1*, Catenin beta-1 gene; DLD, Dyslipidemia; DM, Diabetes; *DNMT3A*, DNA methyltransferase 3 alpha gene; ELISA, Enzyme-linked immunosorbent assay; ERK2, Mitogen-activated protein kinase 1; GCKR, glucokinase regulator; GMPS, Guanine monophosphate synthetase; GNAS, Guanine-nucleotide binding protein subunit-alpha; HCC, Hepatocellular carcinoma; HSP60, Heat shock protein 60; *HSPA5*, Heat shock protein family A member 5 gene; HVASC, Houston Veterans Administration Cirrhosis Surveillance Cohort; HTN, Hypertension; ICGC, International Cancer Gene Consortium; *KMT2C*, Lysine methyltransferase 2C gene; *KMT2D*, Lysine methyltransferase 2D gene; MBOAT7, Membrane-bound O-acyltransferase domain-containing 7; MDA T200.1, MDA Anderson T200.1 Platform; *NFE2L2*, Nuclear factor erythroid 2-like 2 gene; NHS, Normal healthy sera; NHW, Non-Hispanic White; NR, Not reported; *NRAS*, NRAS proto-oncogene GTPase; PCWGC, Pan-Cancer Analysis of Whole Genomes Consortium; PNPLA3, Patatin-like phospholipase domain-containing 3; PRS Polygenic risk score; SERPA, Serum proteome analysis; p16, p16INK4a tumor suppressor protein; *SETDB1*, Set domain bifurcated 1 gene; *TERT*, Telomerase reverse transcriptase gene; THCCC, Texas Hepatocellular Carcinoma Consortium Cohort; TAA, Tumor-associated antigen; TCGA-LIHC, The Cancer Genome Atlas Liver Hepatocellular Carcinoma Cohort; TM6SF2, Transmembrane 6 superfamily member 2; *TP53*, Tumor protein p53 gene.

## Data Availability

No new data were created or analyzed in this study. Data sharing is not applicable to this article.

## Supplementary Materials

The following supporting information can be downloaded at: https://www.mdpi.com/article/10.3390/ijms27104648/s1. Reference [32] is cited in the Supplementary Materials.

## Abbreviations

The following abbreviations are used in this manuscript:

HCC	Hepatocellular carcinoma
MASLD	Metabolic-dysfunction-associated steatotic liver disease
ALD	Alcohol-associated liver disease
IHBD	Intrahepatic bile duct
T2D	Type 2 diabetes mellitus
HCV	Hepatitis C virus
HBV	Hepatitis B virus
MetS	Metabolic syndrome
TCR	Texas Cancer Registry
CCHC	Cameron County Hispanic Cohort
HLCC	Hispanic Liver Cancer Cohort
THCCC	Texas Hepatocellular Carcinoma Consortium Cohort
HVASC	Houston Veterans Administration Cirrhosis Surveillance Cohort
RGV	Rio Grande Valley
RCT	Randomized controlled trial
PRISMA-ScR	Preferred Reporting Items for Systematic Reviews and Meta-Analyses Extension for Scoping Reviews
PNPLA3	Patatin-like phospholipase domain-containing protein 3
